# Preparation of neuroprotective condensed 1,4-benzoxazepines by regio- and diastereoselective domino Knoevenagel–[1,5]-hydride shift cyclization reaction

**DOI:** 10.3762/bjoc.10.272

**Published:** 2014-11-06

**Authors:** László Tóth, Yan Fu, Hai Yan Zhang, Attila Mándi, Katalin E Kövér, Tünde-Zita Illyés, Attila Kiss-Szikszai, Balázs Balogh, Tibor Kurtán, Sándor Antus, Péter Mátyus

**Affiliations:** 1 Department of Organic Chemistry, Semmelweis University, Hőgyes u. 7., 1092 Budapest, Hungary, Fax: +36 1 217-0851; 2 Department of Organic Chemistry, University of Debrecen, Debrecen, P. O. Box 20, 4010 Debrecen, Hungary; 3 CAS Key Laboratory of Receptor Research, Shanghai Institute of Materia Medica, Chinese Academy of Sciences, 555 Zu Chong Zhi Road, Zhang Jiang Hi-Tech Park, Shanghai 201203, PR China; 4 Department of Inorganic and Analytical Chemistry, University of Debrecen, H-4010 Debrecen, Hungary; 5 Porfirin Ltd., Mikszáth K. u. 7. III/3, 4032 Debrecen, Hungary

**Keywords:** 1,4-benzoxazepine, diastereoselective domino Knoevenagel–[1,5]-hydride shift cyclization, neuroprotective, *tert*-amino effect, TDDFT-ECD calculation

## Abstract

Condensed *O*,*N*-heterocycles containing tetrahydro-1,4-benzoxazepine and tetrahydroquinoline moieties were prepared by a regio- and diastereoselective domino Knoevenagel–[1,5]-hydride shift cyclization reaction of a 4-aryl-2-phenyl-1,4-benzoxazepine derivative obtained from flavanone. The relative configuration of products were determined by the correlation of ^3^*J*_H,H_ coupling data with the geometry of major conformers accessed by DFT conformational analysis. Separated enantiomers of the products were characterized by HPLC-ECD data, which allowed their configurational assignment on the basis of TDDFT-ECD calculation of the solution conformers. Two compounds showed neuroprotective activities against hydrogen peroxide (H_2_O_2_) or β-amyloid_25–35_ (Aβ_25–35_)-induced cellular injuries in human neuroblastoma SH-SY5Y cells in the range of those of positive controls.

## Introduction

**Figure 1:**
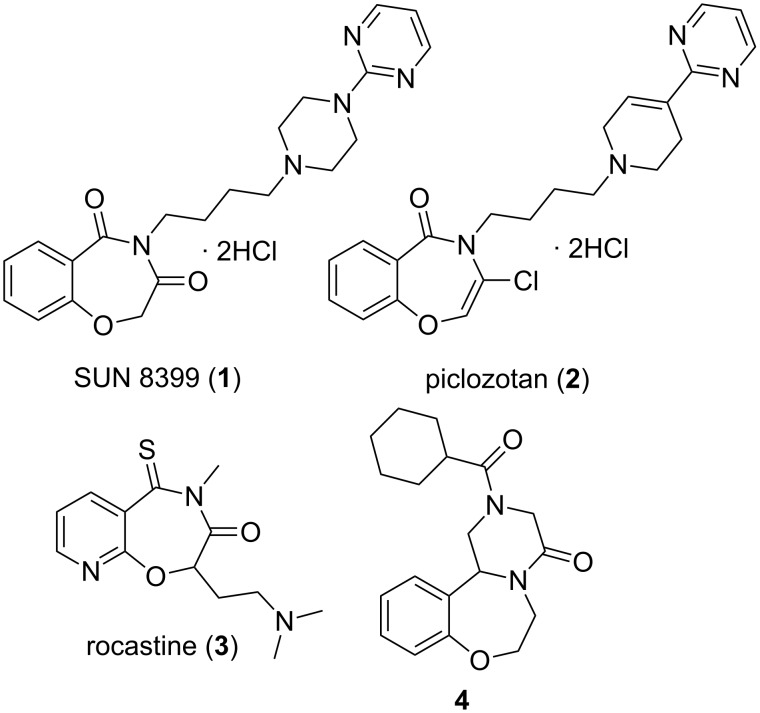
Pharmacologically active derivatives **1**–**4** containing the 1,4-benzoxazepine moiety or its analogue.

The 1,4-benzoxazepine structural unit and its analogues are found in several pharmacologically active derivatives such as the selective 5-HT_1A_ agonist SUN 8399 (**1**) [1], the neuroprotective piclozotan (**2**) [2–3], the antihistaminic rocastine (**3**) [4–5], and the antihelmintic **4** [6] (Figure 1).

**Scheme 1:**
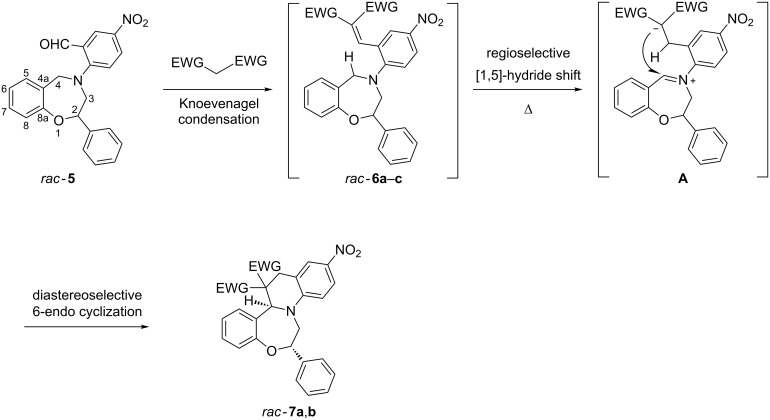
Domino Knoevenagel–[1,5]-hydride shift cyclization reaction for the preparation of condensed 1,4-benzoxazepines.

We report herein the preparation of two neuroprotective condensed 2-phenyl-1,4-benzoxazepines *rac*-**7a**,**b** through the diastereoselective domino Knoevenagel–1,5-hydride shift cyclization reaction *rac*-**5**→*rac*-**7a**,**b** from the readily available 4-aryl-2-phenyl-1,4-benzoxazepine derivative, *rac*-**5** (Scheme 1).

Ring-closure transformations involving C(sp^3^)–H bond functionalization in an internal redox process, particularly involving C–H bonds in α-position to a tertiary amine nitrogen atom, has attracted considerable attention due to its synthetic potential [7–11]. The intramolecular “*tert*-amino effect” induced cyclization consists in (1) the cleavage of the C(sp^3^)–H bond α to a tertiary amine nitrogen through [1,5]-hydride shift (*rac*-**6**→**A**) (Scheme 1) to afford the zwitterionic intermediate **A** and (2) subsequent 6-endo cyclization (**A**→*rac*-**7a**,**b**).

The stereoselective version of “*tert*-amino effect” induced cyclization using chiral metal-catalyzed [12–15] or organocatalytic [16–17] reactions and the mechanism of the stereospecific hydride transfer have been recently studied by several research groups. The synthetic potential of the diastereoselective domino Knoevenagel–[1,5]-hydride shift cyclization reaction was exploited in our present work for the preparation of condensed *O*,*N*-heterocycles with the 1,2,8,9-tetrahydro-7b*H*-quinolino[1,2-*d*][1,4]benzoxazepine skeleton, the neuroprotective activities of which were tested against hydrogen peroxide (H_2_O_2_), Alzheimer's amyloid β-peptide fragment Aβ_25–35_ and oxygen–glucose deprivation (OGD)-induced neurotoxicity in human neuroblastoma SH-SY5Y cells.

## Results and Discussion

**Scheme 2:**
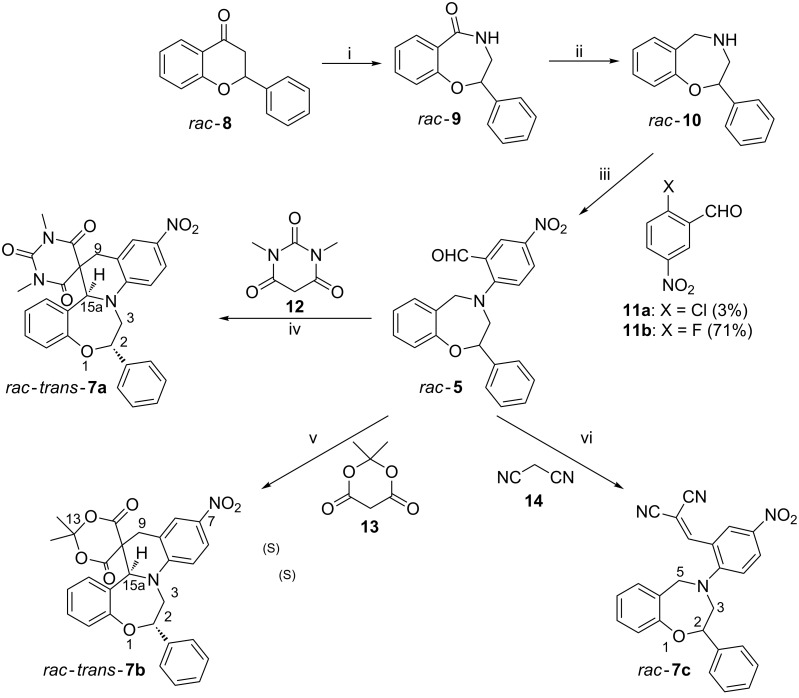
i) a) NaN_3_, CF_3_COOH, b) H_2_O, Δ (77%); ii) LiAlH_4_, dry THF, Δ (80%); iii) **11b**, K_2_CO_3_, toluene, Δ (71%); iv) **12**, MgSO_4_, CHCl_3_ (96%); v) **13**, MgSO_4_, CHCl_3_ (22%); vi) **14**, MgSO_4_, CHCl_3_ (81%).

The starting material 4-aryl-2-phenyl-1,4-benzoxazepine (*rac*-**5**) for the domino Knoevenagel–[1,5]-hydride shift cyclization reaction was prepared from *rac*-flavanone (*rac*-**8**) in four steps (Scheme 2).

The Schmidt reaction of *rac*-**8** was carried out according to the procedure of Litkey and Patonay [18] affording the racemic 3,4-dihydro-2-phenyl-1,4-benzoxazepine-5-one (*rac*-**9**) with high regioselectivity, which was reduced to (*rac*)-**10** with LAH in dry THF. In the following step, the *N*-arylation of *rac*-**10** was performed through a nucleophilic aromatic substitution by using 2-fluoro-5-nitrobenzaldehyde (**11b**) to give *rac*-**5** containing a tertiary arylamine nitrogen in 71% yield. With the 2-chloro-5-nitrobenzaldehyde reagent, only 3% yield for *rac*-**5** could be achieved. Subsequently *rac*-**5** was reacted with 1,3-dimethylbarbituric acid (**12**), Meldrum’s acid (**13**) and malononitrile (**14**) all containing active methylene groups, which initiated a domino reaction (Scheme 2). The Knoevenagel reaction of the formyl group in *rac*-**5** and the active methylene groups of **12**–**14** afforded the intermediates *rac*-**6a**–**c** (Scheme 1), which underwent regioselective [1,5]-hydride shift with participation of the benzylic hydrogen to result in the zwitterionic iminium ion intermediates **A**. The 6-endo cyclization of this intermediate gave *rac*-*trans*-**7a**,**b** with high diastereoselectivity, which was governed by the C-2 chirality center of intermediate **A**. In contrast to the domino reaction with 1,3-dimethylbarbituric acid (**12**) and Meldrum’s acid (**13**), the reaction with malonitrile (**14**) stopped at the stage of the Knoevenagel product *rac*-**7c** with MgSO_4_ in CHCl_3_ and further cyclization did not occur when heated in DMSO at 150 °C or refluxed in *n*-butanol.

**Figure 2:**
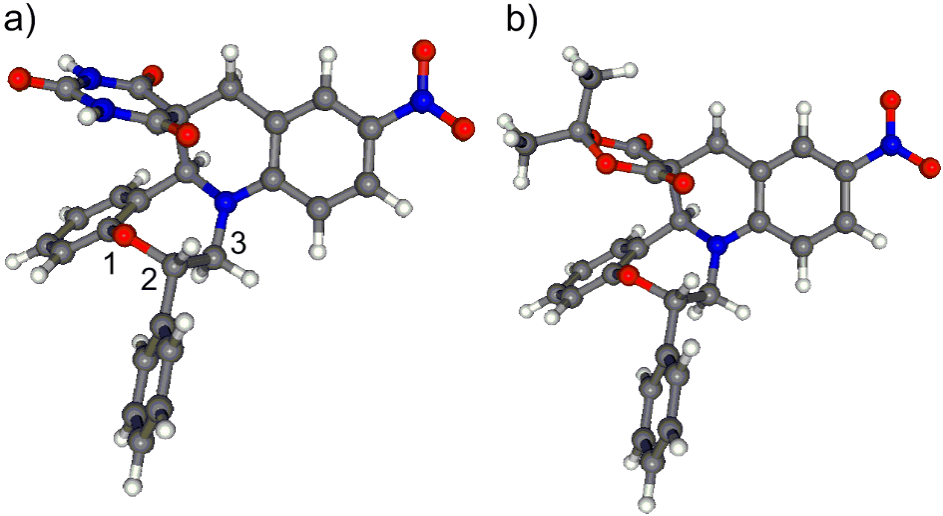
Lowest-energy conformers of a) *trans*-(2*S*,15a*S*)-**7a** (>99.9%) with the replacement of the *N*-methyl groups by hydrogen atoms; b) *trans*-(2*S*,15a*S*)-**7b** (97.9%) obtained by the B3LYP/6-31G(d) reoptimization of the initial MMFF conformers. Boltzmann weights for the same conformers in the PCM calculations are 99.8% and 95.8%, respectively.

**Figure 3:**
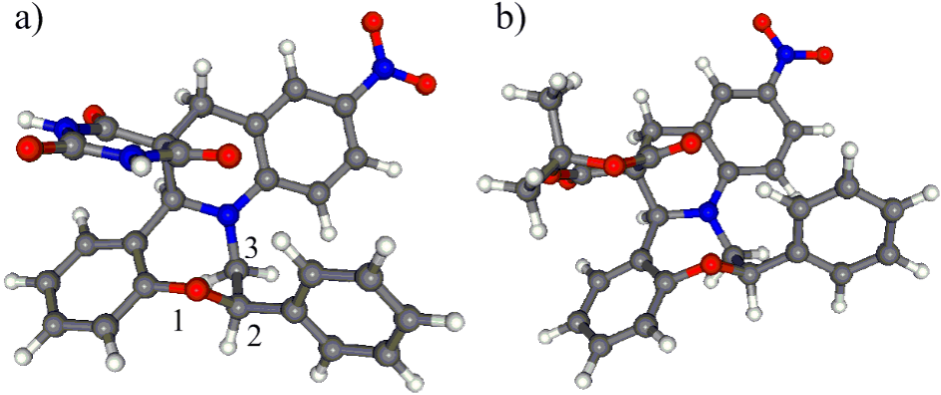
Lowest-energy conformers of a) *cis*-(2*R*,15a*S*)-**7a** (99.4%) with the replacement of the *N*-methyl groups by hydrogen atoms; b) *cis*-(2*R*,15a*S*)-**7b** (84.5%) obtained by the B3LYP/6-31G(d) reoptimization of the initial MMFF conformers. Boltzmann weights for the same conformers in the PCM calculations are 97.7% and 82.5%, respectively.

The NMR data of **7a**,**b** clearly indicated that they were single diastereomers and the determination of the relative configuration was carried out by the correlation of NMR data and DFT conformational analysis of *cis*- and *trans*-**7a**,**b** (Figure 2 and Figure 3).

Although the hydrogens 2-H and 15a-H of *cis*-**7a**,**b** are expected to point toward the same side of the molecules, the analysis of the computed solution conformers of *cis*-**7a**,**b** revealed that the orientation and distance (>4.6 Å) of these methine protons (Figure 3) does not allow for the detection of their NOE contact, rendering the NOE-based assignment of the relative configuration ambiguous. Similarly, no NOE correlation is expected between 2-H and 15a-H of *trans*-**7a**,**b**, which was also evident from the structures of their computed lowest-energy conformers (Figure 2). Accordingly, no characteristic NOE was observed between these methine protons for **7a**,**b**, which did not help for the assignment of the relative configuration. However, in the computed solution conformers of *cis*-**7a**,**b**, the oxazepine ring adopts a twist boat conformation with axial 2-H showing *gauche* orientation with adjacent 3-H protons (e.g., ω_2-Hax,C-2,C-3,3-Hax_ ≈ 36.2°, ω_2-Hax,C-2,C-3,3-Heq_ ≈ −79.2° in **7a**), suggesting small ^3^*J*_2-H,3-H_ couplings.

Whereas in the computed solution conformers of *trans*-**7a**,**b** (Figure 2), the oxazepine ring adopts a boat conformation showing a *trans-*diaxial relationship with the adjacent 3-H_ax_. In accordance with the calculations, the measured values of the ^3^*J*_2-Hax,3-H_ (11.5 and 3.2 Hz) corroborate that the couplings between 2-H_ax_ and 3-Hs derive from *trans-*diaxial (ω_2-Hax,C-2,C-3,3-Hax_ ≈ 180°) and *gauche* (ω_2-Hax,C-2,C-3,3-Heq_ ≈ 60°) orientations, which is only feasible with the *trans* relative configuration of **7a**,**b**. Thus in the course of the cyclization, the attack of the carbanion to the iminium carbon of intermediate **A** occurred with *trans*-diastereoselectivity to the C-2 phenyl group. The formation of the diastereomeric *cis*-**7a**,**b** could not be detected.

**Figure 4:**
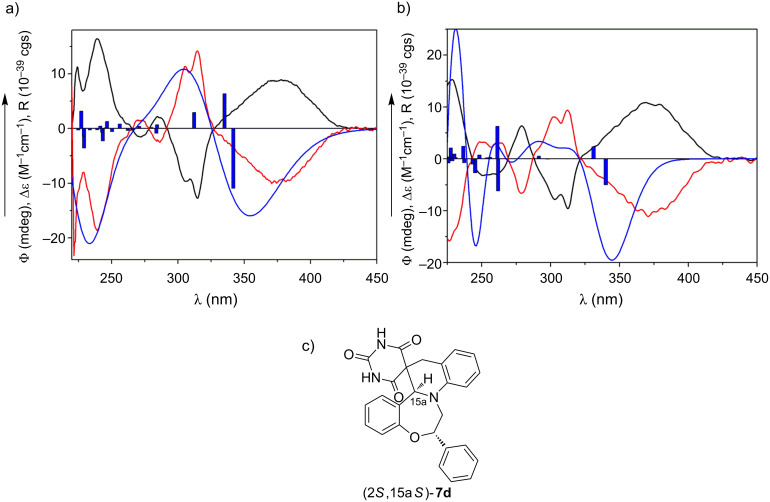
HPLC-ECD spectra of the first-eluting (black curve) and second-eluting (red curve) enantiomers of a) *trans*-**7a**, and b) *trans*-**7b** compared with the computed ECD spectra of the gas-phase optimized conformers (>1%) of model compound (2*S*,15a*S*)-**7d** (1 conformer, B3LYP/TZVP) and (2*S*,15a*S*)-**7b** (2 conformers, BH&HLYP/TZVP). Bars represent the computed rotational strength values (R/8) of the lowest-energy conformer. c) Structure of the (2*S*,15a*S*)-**7d** model compound of *trans*-**7a** used for the conformational analysis and ECD calculations.

The chiral HPLC analysis of the products **7a**,**b** also confirmed that the reaction took place diastereoselectively and enantiomers of *trans*-**7a**,**b** were separated on a Chiralpak IA column using hexane/dichloromethane as eluent and online HPLC-ECD spectra of the separated enantiomers were recorded. Due to their similar chromophoric system, the HPLC-ECD spectra of **7a** and **7b** were quite similar. The first-eluting enantiomer of **7a** had an intense broad positive Cotton effect (CE) at 378 nm, negative ones at 314, 305, 272 nm and positive ones at 284, 279 and 224 nm (Figure 4a).

The HPLC-ECD spectrum of the first-eluting enantiomer of **7b** showed similar ECD pattern with somewhat different shape and intensities in the 290–240 nm range (Figure 4b). TDDFT-ECD calculation was proved an efficient method to determine the absolute configuration of separated stereoisomers of bioactive synthetic [19] and natural derivatives [20–21] on the basis of their HPLC-ECD spectra.

For the configurational assignment of the separated enantiomers, TDDFT-ECD calculations were carried out on the solution conformers of (2*S*,15a*S*)-**7d** (*N*-methyl groups were replaced by hydrogens for the calculation) and (2*S*,15a*S*)-**7b** and computed ECD curves were compared with the measured HPLC-ECD ones of the separated enantiomers of *trans*-**7a**,**b** (Figure 4a and Figure 4b). The computed TDDFT-ECD spectra of (2*S*,15a*S*)-**7d** and (2*S*,15a*S*)-**7b** gave good agreement with the experimental HPLC-ECD spectra of the second-eluting enantiomers (Figure 4a,b), which allowed determining the absolute configuration of their second-eluting enantiomers (negative CEs at 370 and 371 nm, respectively) as (2*S*,15a*S*). The good agreement between the experimental HPLC-ECD and computed ECD spectra of **7a**,**b** not only allowed the configurational assignment of their separated enantiomers but also confirmed independently the *trans*-diastereoselectivity of the cyclization.

**Figure 5:**
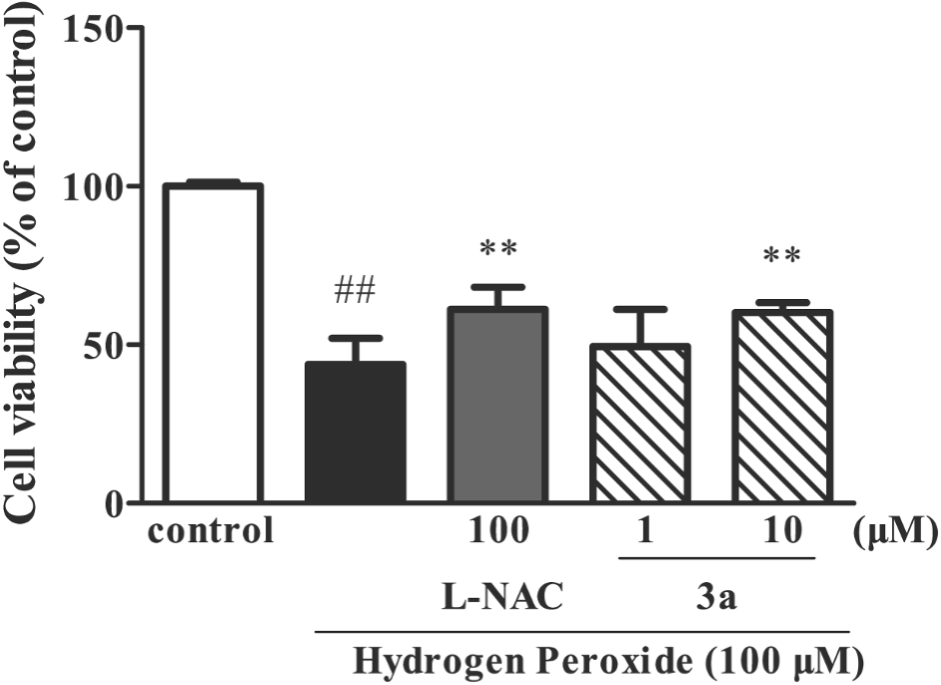
Protective effect of compound **7a** on hydrogen peroxide-induced neurotoxicity in SH-SY5Y cells. ^##^P < 0.01 vs control group, **P< 0.01 vs hydrogen peroxide group.

**Figure 6:**
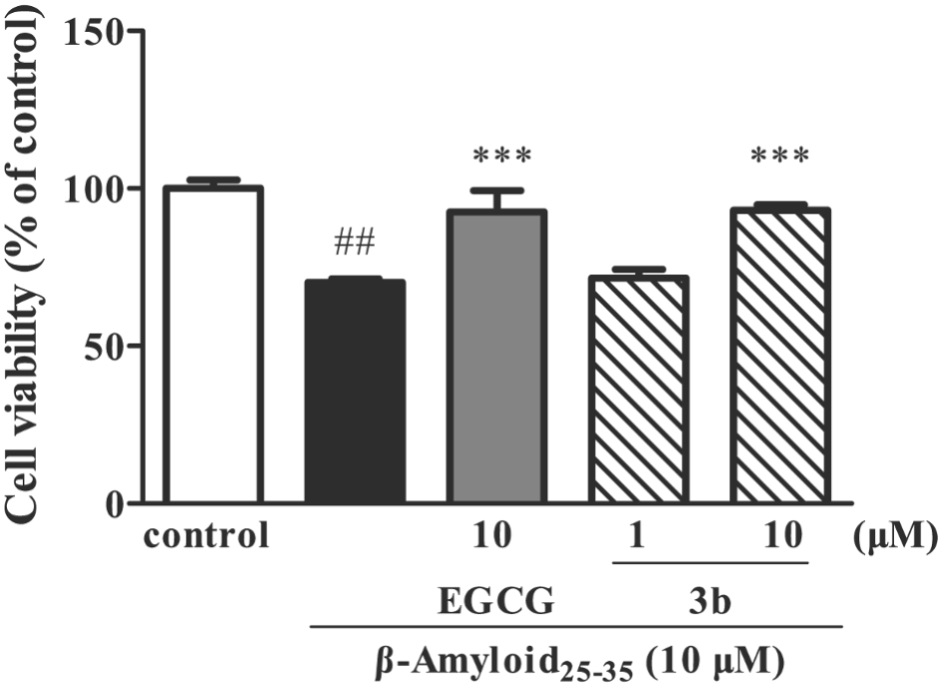
Protective effect of compound **7b** on β-amyloid_25–35_-induced neurotoxicity in SH-SY5Y cells. ^##^P < 0.01 vs control group, **P< 0.01 vs β-amyloid_25–35_ group.

The neuroprotective activities of *rac*-**7a**,**b** and *rac*-**5** were tested against hydrogen peroxide (H_2_O_2_), β-amyloid-25-35 (Aβ_25–35_) and oxygen–glucose deprivation (OGD)-induced neurotoxicity in human neuroblastoma SH-SY5Y cells [22]. The preliminary screenings showed that *rac*-**7a** at 10 µM concentration displayed neuroprotective activity against H_2_O_2_-induced cellular injuries in human neuroblastoma SH-SY5Y cells with 16.4% increase in cell viability (Figure 5), while 10 µM of *rac*-**7b** increased cell viability in Aβ_25–35_-induced neurotoxicity by 22.8% (Figure 6).

The positive controls with *N*-L-acetylcysteine (NAC) [23] and epigallocatechin gallate (EGCG) [24] increased cell viability by 17.3% and 22.4% in H_2_O_2_ and Aβ_25–35_ model, respectively. Compounds *rac*-**7a**,**b** and *rac*-**5** did not show acetylcholinesterase inhibitory effect.

## Conclusion

Regioselective domino Knoevenagel–[1,5]-hydride shift cyclization reactions of a 4-aryl-2-phenyl-1,4-benzoxazepine derivative afforded three condensed *O*,*N*-heterocycles containing tetrahydro-1,4-benzoxazepine and tetrahydroquinoline moieties. *trans*-Diastereoselectivity of the cyclizations was determined by the correlation of ^3^*J*_H,H_ coupling constants with the geometry of the computed conformers, while (2*R*,15a*R*) absolute configurations of **7a**,**b** were assigned to the separated enantiomers showing positive CE for the lowest-energy ECD transition by the comparison of the experimental HPLC-ECD spectra with those obtained by the TDDFT-ECD calculations of the solution conformers. Compound **7a** showed neuroprotective activity against hydrogen peroxide (H_2_O_2_), while **7b** against β-amyloid_25–35_ (Aβ_25–35_)-induced cellular injuries in human neuroblastoma SH-SY5Y cells with 16.4% and 22.8% increase in cell viability at 10 μM concentrations, respectively.

## Experimental

Melting points were determined on a Kofler hot-stage apparatus and are uncorrected. The NMR spectra were recorded on Bruker-AMX 500 (^1^H: 500 MHz; ^13^C: 125 MHz) and Bruker Avance II 400 (^1^H: 400 MHz; ^13^C: 100 MHz) spectrometers using TMS as internal standard. Chemical shifts were reported as δ in ppm and ^3^*J*_H,H_ coupling constants in Hz. Chiral HPLC separation of **7a**,**b** were performed on a Jasco HPLC system with Chiralpak IA column (5 μm, 150 × 4.6 mm, hexane/dichloromethane 80:20 or 70:30 eluents, 1 mL min^−1^ flow rate) and HPLC-ECD spectra were recorded in stopped-flow mode on a JASCO J-810 electronic circular dichroism spectropolarimeter equipped with a 10 mm HPLC flow cell. ECD ellipticity (Φ) values were not corrected for concentration. For an HPLC-ECD spectrum, three consecutive scans were recorded and averaged with 2 nm bandwidth, 1 s response, and standard sensitivity. The HPLC-ECD spectrum of the eluent recorded in the same way was used as background. The concentration of the injected sample was set so that the HT value did not exceed 500 V in the HT channel down to 230 nm. IR spectra were recorded on a JASCO FTIR-4100 spectrometer and absorption bands are presented as wavenumber in cm^−1^. Electrospay Quadrupole Time-of-Flight HRMS measurements were performed with a MicroTOF-Q type QqTOF MS instrument equipped with an ESI source from Bruker (Bruker Daltoniks, Bremen, Germany). Elementary analysis was carried with a Vario Micro V1.9.6 instrument.

**Computational section:** Mixed torsional/low mode conformational searches were carried out by means of the Macromodel 9.9.223 software [25] using OPLS-2005 force field with implicit solvent model for chloroform applying a 42 kJ/mol energy window. Geometry reoptimizations (B3LYP/6-31G(d) level in gas phase and B3LYP/TZVP level with PCM solvent model for CHCl_3_) and TDDFT-ECD calculations were performed with Gaussian 09 [26] using various functionals (B3LYP, BH&HLYP, PBE0) and TZVP basis set for ECD calculations. ECD spectra were generated as the sum of Gaussians [27] with 2100, 2400 and 4200 cm^−1^ half-height width (corresponding to ca. 19, 22 and 38 nm at 300 nm) using dipole-velocity computed rotational strengths. Boltzmann distributions were estimated from the ZPVE-corrected B3LYP/6-31G(d) energies in the gas-phase calculations and from the B3LYP/TZVP energies in the PCM ones. The MOLEKEL [28] software package was used for visualization of the results.

**Bioassay on neuroprotective activity**: SH-SY5Y cells were high passages from the American Type Culture Collection and were maintained at 37 °C in a humidified atmosphere containing 5% CO_2_. Cells were pretreated with compounds for 2 h and then suffered cell injury by treatment with 10 µM Aβ_25–35_ or 100 µM H_2_O_2_ for another 24 h; to SH-SY5Y cells, pretreated with compounds before exposed to OGD for 1 h, was added 1 mg/mL glucose and 10% serum and cultured for another 24 h under normal condition. SH-SY5Y cells cultured with glucose under normal condition served as control. Cell viability was evaluated by incubating cells with 0.5 mg/mL 3-[4,5-dimethylthiazol-2-yl]-2,5-diphenyltetrazolium bromide (MTT) for 3 h under 5% CO_2_/95% air at 37 °C. After replacement of the medium with 100 µL DMSO, absorbance was read at 490 nm. Data were analyzed by one-way analysis of variance (ANOVA) and expressed as means ± SD with P < 0.05 as significance.

**2-Phenyl-3,4-dihydro-1,4-benzoxazepin-5(2*H*)-one (*rac*-9):** To a stirred solution of racemic flavanone (*rac***-8**, 10.00 g, 44.63 mmol) in TFA (55 mL), sodium azide (5.80 g, 89.22 mmol) was added in two parts and the mixture was stirred for 6 h. Cold diethyl ether (200 mL) was added to the reaction mixture and stirring was continued for 1 h. The precipitated white solid was filtered and refluxed in 180 mL water for 3 h. After cooling to room temperature, it was refrigerated for 1 h, the resultant precipitate was filtered and washed with cold water. The product *rac*-**9** was dried and isolated as white powder (8.23 g, 77%) with mp 127–129 °C. ^1^H NMR (400 MHz, CDCl_3_) δ 3.54 (m, 3-H_a_, 1H), 3.66 (m 3-Hb, 1H), 5.46 (dd, *J* = 9.7 and 3.6 Hz, 2-H, 1H), 7.07 (d, *J* = 8.4 Hz, 9-H, 1H), 7.19 (m, *J* = 7.6 Hz, 7-H, 1H), 7.28 (bs, NH, 1H), 7.36–7.44 (m, Ph, 5H), 7.50 (m, *J* = 7.6 Hz, 8-H, 1H), 7.85 (dd, *J* = 8.0 and 1.6 Hz, 6-H, 1H); ^13^C NMR (100 MHz, CDCl_3_) δ 46.3 (C-3), 85.8 (C-2), 122.4 (C-9), 123.7 (C-7), 125.9 (C-5a), 126.3 (C-2’, C-6’), 128.5 (C-4’), 128.7 (C-3’, C-5’), 130.9 (C-6), 133.3 (C-8), 139.0 (C-1’), 154.5 (C-9a), 170.9 (C-5); IR (KBr) ν: 1462, 1665, 2917, 3075, 3203, 3307 cm^−1^; HRMS–ESI (*m*/*z*): [M + Na]^+^ calcd for C_15_H_13_NO_2_Na, 262.0844; found, 262.0839; Anal. calcd for C_15_H_13_NO_2_ (239.09): C, 75.30; H, 5.48; N, 5.85; found: C, 75.28; H, 5.49; N, 5.85.

**2-Phenyl-2,3,4,5-tetrahydro-1,4-benzoxazepine (*rac*-10):** To a stirred solution of *rac*-**9** (8.13 g, 33.98 mmol) in dry THF (80 mL), 2.0 M lithium aluminium hydride solution in THF was added dropwise (10 mL, 0.76 g, 20.03 mmol) and the mixture was refluxed for 1.5 h. After cooling to room temperature, ethyl acetate (5 mL), methanol (5 mL) and water (50 mL) were added and the mixture was concentrated under reduced pressure. The residue was extracted with dichloromethane (3 × 50 mL). The combined organic layers were washed with water (20 mL), dried over MgSO_4_, filtered and concentrated under reduced pressure. The product *rac*-**10** was isolated as yellow solid (6.12 g, 80%) with mp 73–74 °C. ^1^H NMR (400 MHz, CDCl_3_) δ 3.20 (dd, *J* = 14.4 and 10.0 Hz, 3-H_ax_, 1H), 3.37 (d, 3-H_eq_, 14.4 Hz, 1H), 3.96 (d, *J* = 14.8 Hz, 5-H_a_, 1H), 4.12 (d, *J* = 14.8 Hz, 5-H_b_, 1H), 4.66 (d, *J* = 10.0 Hz, 2-H, 1H), 7.04 (m, 7-H, 8-H, 2H), 7.17 (m, 6-H, 9-H, 2H), 7.31–7.43 (m, Ph, 5H); ^13^C NMR (100 MHz, CDCl_3_) δ 52.5 (C-5), 58.9 (C-3), 86.4 (C-2), 121.6 (C-9), 123.6 (C-7), 125.8 (C-2’, C-6’), 127.7 (C-8), 128.4 (C-3’, C-5’), 129.0 (C-4’, C-6), 135.6 (C-5a), 140.5 (C-1’), 159.1 (C-9a); IR (KBr) ν: 1579, 2935, 3328 cm^−1^; HRMS–ESI (*m*/*z*): [M + H]^+^ calcd for C_15_H_16_NO, 226.1232; found, 226.1235; Anal. calcd for C_15_H_15_NO (225.11): C, 79.97; H, 6.71; N, 6.22; found: C, 80.03; H, 6.76; N, 6.18.

**5-Nitro-2-(2-phenyl-2,3-dihydro-1,4-benzoxazepin-4(5*H*)-yl)benzaldehyde (*rac*-5):** To the stirred solution of *rac*-**10** (1.50 g, 6.66 mmol) in dry toluene (25 mL), anhydrous K_2_CO_3_ (1.85 g 13.39 mmol) and 2-fluoro-5-nitrobenzaldehyde (1.35 g, 7.98 mmol) were added and the mixture was refluxed for 8 h. After cooling to room temperature, K_2_CO_3_ was filtered off and toluene was removed under reduced pressure. The residue was purified by column chromatography on silica gel (ethyl acetate/hexane 4:1) to give *rac*-**5** as yellow solid (1.77 g, 71%). Mp 152–156 °C; ^1^H NMR (400 MHz, CDCl_3_) δ 3.63 (dd, *J* = 10.8 and 13.6 Hz, 3-H_ax_, 1H), 4.04 (dd, *J* = 13.6 and 2.4 Hz, 3-H_eq_, 1H), 4.71 (d, *J* = 16.4 Hz, 5-H_a_, 1H), 4.94 (d, *J* = 16.4 Hz, 5-H_b_, 1H), 5.29 (dd, *J* = 10.8 and 2.4 Hz, 2-H, 1H), 7.03 (m, *J* = 9.6 Hz, 6-H, 9-H, 2H), 7.13 (m, 8-H, 1H), 7.26 (m, 6”-H, 7-H, 2H), 7.36–7.42 (m, Ph, 5H), 8.17 (dd, *J* = 9.2 Hz and 2.8 Hz, 5”-H, 1H), 8.61 (d, *J* = 2.8 Hz, 3”-H, 1H), 9.97 (s, CHO, 1H); ^13^C NMR (100 MHz, CDCl_3_) δ 56.1 (C-5), 64.6 (C-3), 82.7 (C-2), 117.6 (C-6”), 121.0 (C-9), 123.8 (C-7), 124.3 (C-5a), 125.9 (C-2’, C-6’), 127.5 (C-2’’), 128.1 (C-4”), 128.7 (C-8, C-3”), 129.1 (C-3’, C-5’), 129.5 (C-5”), 129.8 (C-4’), 129.8 (C-6), 138.1 (C-1’), 156.8 (C-9a), 158.2 (C-1”), 188.3 (CHO); IR (KBr) ν: 1382, 1500, 1681, 1734, 2921 cm^−1^; HRMS–ESI (*m*/*z*): [M + Na]^+^ calcd for C_22_H_18_N_2_O_4_Na, 397.1164; found, 397.1158; Anal. calcd for C_22_H_18_N_2_O_4_ (374.12): C, 70.58; H, 4.85; N, 7.48; found: C, 70.59; H, 4.89; N, 7.45.

***trans-*1,3-Dimethyl-11'-nitro-2'-phenyl-1',2'-dihydro-2*H*,7b'*H*,9'*H*-spiro[pyrimidine-5,8'-quinolino[1,2-*d*][1,4]benzoxazepine]-2,4,6(1*H*,3*H*)-trione (*rac*-*trans-*7a):** To a stirred solution of *rac*-**5** (100 mg, 0.27 mmol) in chloroform (5 mL), anhydrous MgSO_4_ (150 mg, 1.25 mmol) and 1,3-dimethylbarbituric acid (60 mg, 0.38 mmol) were added and the mixture was refluxed for 4 h. After cooling to room temperature, MgSO_4_ was filtered off and chloroform was removed under reduced pressure. Water (10 mL) and dichloromethane (20 mL) were added and the layers were separated. The aqueous phase was extracted with dichloromethane (2 × 10 mL). The combined organic layers were washed with concentrated NaHCO_3_ solution, dried over MgSO_4_, filtered and concentrated under reduced pressure. The oily product was triturated with ether to afford *rac*-*trans-***7a** as yellow solid (131 mg, 96%) with mp 262–264 °C. ^1^H NMR (400 MHz, CDCl_3_) δ 2.91 (s, N-Me, 3H), 3.12 (d, *J* = 16.8 Hz, 9-H_a_, 1H), 3.13 (s, N-Me, 3H), 3.87 (dd, *J* = 16.4 and 12.0 Hz, 3-H_ax_, 1H), 4.00 (d, *J* = 16.8 Hz, 9-H_b_, 1H), 4.16 (dd, *J* = 12.0 and 3.2 Hz, 3-H_eq_, 1H), 4.81 (s, 15a-H, 1H), 5.13 (dd, *J* = 12.0 and 3.2 Hz, 2-H, 1H), 6.80 (d, *J* = 7.6 Hz, 19-H, 1H), 6.85 (d, *J* = 9.2 Hz, 5-H, 1H), 7.10 (m, 16-H, Ph, 3H), 7.15 (m, 17-H, 1H), 7.30–7.38 (m, 18-H, Ph, 4H), 8.08 (dd, *J* = 9.2 and 2.4 Hz, 6-H, 1H), 8.13 (s, 8-H, 1H); ^13^C NMR (100 MHz, CDCl_3_) δ 28.7 (Me), 28.9 (Me), 34.7 (C-9), 47.3 (C-3) 51.9 (C-10), 71.5 (C-15a), 81.2 (C-2), 109.9 (C-19), 121.5 (C-15b), 123.8 (C-5), 124.7 (C-17), 124.8 (C-6), 125.5 (C-8a), 127.0 (C-2’ and C-6’), 128.8 (C-3’ and C-5’), 129.1 (C-8), 129.9 (C-18), 132.1 (C-16), 137.6 (C-7), 138.3 (C-1’), 147.6 (C-13), 150.1 (C-19a), 151.7 (C-4a), 167.1 (C-15), 168.9 (C-11); IR (KBr) ν: 749, 988, 1326, 1683, 2926 cm^−1^; HRMS–ESI (*m*/*z*): [M + Na]^+^ calcd for C_28_H_24_N_4_O_6_Na, 535.1594; found, 535.1588; Anal. calcd for C_28_H_24_N_4_O_6_ (512.17): C, 65.65; H, 4.72; N, 10.93; found: C, 65.64; H, 4. 70; N, 10.95. (2*R*,15a*R*)-**7a**: retention time (*t*_R_) 10.57 min (Chiralpak IA, hexane/dichloromethane 70:30); HPLC-ECD data in hexane/dichloromethane 70:30 as λ_max_ (Φ): 378 (8.97), 368 sh (8.71), 314 (−12.73), 305 sh (−12.73), 284 (2.21), 272 (−1.54), 239 (16.42), 224 sh (11.27). (2*S*,15a*S*)-**7a**: retention time (*t*_R_) 28.73 min (Chiralpak IA, hexane/dichloromethane 70:30**)**; HPLC-ECD in hexane/dichloromethane 70:30 as λ_max_ (Φ): 381 sh (−4.83), 370 (−5.03), 314 (7.10), 305 sh (5.68), 286 (−1.24), 270 (0.78), 239 (−9.32), 221 (−11.60).

***trans-*2,2-Dimethyl-11'-nitro-2'-phenyl-1',2'-dihydro-7b'*H*,9'*H*-spiro[1,3-dioxane-5,8'-quinolino[1,2-*d*][1,4]benzoxazepine]-4,6-dione (*rac*-*trans-*7b):** To the stirred solution of *rac*-**5** (100 mg, 0.27 mmol) in chloroform (5 mL), anhydrous MgSO_4_ (150 mg, 1.25 mmol) and Meldrum’s acid (54 mg, 0.31 mmol) were added and the mixture was refluxed for 6 h. After cooling to room temperature, MgSO_4_ was filtered off and chloroform was removed under reduced pressure. Water (10 mL) and dichloromethane (20 mL) were added and the layers were separated. The aqueous phase was extracted with dichloromethane (2 ×10 mL). The combined organic layers were washed with concentrated NaHCO_3_ solution, dried over MgSO_4_, filtered and concentrated under reduced pressure. The oily product was triturated with ether to afford *rac*-*trans-***7b** as yellow solid (30 mg, 22%) with mp 130–134 °C. ^1^H NMR (500 MHz, CDCl_3_) δ 1.06 (s, Me, 3H), 1.62 (s, Me, 3H), 3.19 (d, *J* = 16.5 Hz, 9-H_a_, 1H), 3.90 (d, *J* = 16.5 Hz, 9-H_b_, 1H), 3.95 (dd, *J* = 16.5 and 11.5 Hz, 3-H_ax_, 1H), 4.16 (dd *J* = 16.5 Hz and 3.2 Hz, 3-H_eq_, 1H), 5.02 (s, 15a-H, 1H), 5.22 (dd, *J* = 11.5 Hz and 3.2 Hz, 2-H, 1H), 6.83 (d, *J* = 8.0 Hz, 19-H, 1H), 6.87 (d, *J* = 9.0 Hz, 5-H, 1H), 7.12 (m, 16-H, 1H), 7.22 (m, 17-H, 18-H, 2H), 7.27–7.35 (m, Ph, 5H), 8.09 (m, 8-H, 6-H, 2H); ^13^C NMR (100 MHz, CDCl_3_) δ 26.9 (Me), 30.5 (Me), 35.7 (C-9), 47.2 (C-3), 50.0 (C-10), 70.7 (C-15a), 80.3 (C-2), 105.5 (C-13), 110.0 (C-19), 119.5 (C-8a), 125.1 (C-5), 125.2 (C-17), 125.6 (C-6), 126.7 (C-15b), 127.2 (C-2’, C-6’), 128.6 (C-3’, C-5’), 129.0 (C-8), 131.1 (C-4’), 131.4 (C-16), 137.3 (C-7), 138.1 (C-1’), 147.7 (C-19a), 152.2 (C-4a), 164.5 (C-15), 168.2 (C-11); IR (KBr) ν: 1197, 1322, 1507, 1737, 2925 cm^−1^; HRMS–ESI (*m*/*z*): [M + Na]^+^ calcd for C_28_H_24_N_2_O_7_Na, 523.1481; found, 523.1475; Anal. calcd for C_28_H_24_N_2_O_7_ (500.16): C, 67.19; H, 4.83; N, 5.60; found: C, 67.17; H, 4.86; N, 5.59. (2*R*,15a*R*)-**7b**: retention time (*t*_R_) 5.96 min (Chiralpak IA, hexane/dichloromethane 70:30**)**; HPLC-ECD data in hexane/dichloromethane 70:30 as λ_max_ (Φ): 379 sh (10.62), 368 (10.90), 312 (−9.79), 303 sh (−7.89), 279 (6.49), 261 sh (−3.01), 251 (−3.30), 228 (15.44). (2*S*,15a*S*)-**7b**: retention time (*t*_R_) 14.09 min (Chiralpak IA, hexane/dichloromethane 70:30**)**; HPLC-ECD data in hexane/dichloromethane 70:30 as λ_max_ (Φ): 383 sh (−3.29), 371 (−3.71), 311 (3.16), 302 sh (2.75), 278 (−2.26), 260 sh (1.04), 249 (1.10), 227 (−5.51).

**[5-Nitro-2-(2-phenyl-2,3-dihydro-1,4-benzoxazepin-4(5*H*)-yl)benzylidene]propanedinitrile (7c):** To the stirred solution of *rac*-**5** (100 mg, 0.27 mmol) in chloroform (5 mL), anhydrous MgSO_4_ (150 mg, 1.25 mmol) and malononitrile (130 mg, 1.97 mmol) were added and the mixture was refluxed for 10 h. After cooling to room temperature, MgSO_4_ was filtered off and chloroform was removed under reduced pressure. Water (10 mL) and dichloromethane (20 mL) were added and the layers were separated. The aqueous phase was extracted with dichloromethane (2 × 10 mL). The combined organic layers were washed with concentrated NaHCO_3_ solution, dried over MgSO_4_, filtered and concentrated under reduced pressure. The oily product was triturated with ether to afford **7c** as yellow solid (91 mg, 81%) with mp 152–156 °C. ^1^H NMR (400 MHz, CDCl_3_) δ 3.78 (m, 3-H, 2H), 4.50 (d, *J* = 15.6 Hz, 11-H_a_, 1H), 4.58 (d, *J* = 15.6 Hz, 11-H_b_, 1H), 5.06 (dd, *J* = 10.0 Hz and 2.4 Hz, 3-H_eq_, 1H), 7.15 (d, *J* = 8.0 Hz, 15-H, 1H), 7.16–7.26 (m, 12-H, 13-H, 14-H, 3H), 7.32 (m, 7-H, 1H), 7.41 (m, Ph, 5H), 7.75 (s, 16-H, 1H), 8.26 (dd, *J* = 9.2 Hz and 2.6 Hz, 7-H, 1H), 8.85 (d, *J* = 2.6 Hz, 9-H, 1H); ^13^C NMR (100 MHz, CDCl_3_) δ 59.4 (C-11), 63.2 (C-3), 82.9 (C-2), 84.3 (C-17), 111.8 (CN), 112.9 (CN), 119.2 (C-15), 121.5 (C-6), 121.9 (C-10), 124.5 (C-13), 125.9 (C-2’, C-6’, C-9), 128.4 (C-11a), 128.7 (C-7) 128.9 (C-3’, C-5’, C-14), 129.2 (C-4’), 129.9 (C-12), 138.2 (C-8), 141.2 (C-1’), 156.9 (C-16), 158.2 (C-15a), 158.5 (C-5) IR (KBr) ν: 759, 1222, 1335, 1488, 2227, 2923 cm^−1^; HRMS–ESI (*m*/*z*): [M+Na]^+^ calcd for C_25_H_18_N_4_O_3_Na, 445.1277; found, 445.1271; Anal. calcd for C_28_H_18_N_4_O_3_ (422.14): C, 71.08; H, 4.29; N, 13.26; found: C, 71.05; H, 4.30; N, 13.25. First eluting enantiomer of **7c**: retention time (*t*_R_) 21.41 min (Chiralpak IA, hexane/dichloromethane 80:20); HPLC-ECD data in hexane/dichloromethane 80:20 as λ_max_ (Φ): 348 (−4.21), 279 (3.84), 252 sh (1.62), 223 (2.04). Second eluting enantiomer of **7c**: retention time (*t*_R_) 21.41 min (Chiralpak IA, hexane/dichloromethane 80:20); HPLC-ECD data in hexane/dichloromethane 80:20 as λ_max_ (Φ): 356 (3.97), 281 (−3.48), 250 sh (−1.32).

## Supporting Information

File 1 Spectroscopic data and details of calculations.
Structures and populations of the in vacuo and PCM solvent model conformers, cartesian coordinates of **7b–d**, ^1^H, ^13^C NMR and IR spectra are disclosed in this file.

## References

[1] Kuribara H (1994). Jpn J Pharmacol.

[2] Kamei K, Maeda N, Nomura K, Shibata M, Katsuragi-Ogino R, Koyama M, Nakajima M, Inoue T, Ohno T, Tatsuoka T (2006). Bioorg Med Chem.

[3] Tani Y, Ogata A, Koyama M, Inoue T (2010). Eur J Pharmacol.

[4] Nolan J C, Stephens D J, Proakis A G, Leonard C A, Johnson D N, Kilpatrick B F, Foxwell M H, Yanni J M (1989). Agents Actions.

[5] Sleevi M C, Cale A D, Gero W T, Jaques L W, Welstead W J, Johnson A F, Kilpatrick B F, Demian I, Nolan J C, Jenkins H (1991). J Med Chem.

[6] Brewer M D, Burgess M N, Dorgan R J J, Elliott R L, Mamalis P, Manger B R, Webster R A B (1989). J Med Chem.

[7] Meth-Cohn O, Suschitzky H (1972). Adv Heterocycl Chem.

[8] Meth-Cohn O (1996). Adv Heterocycl Chem.

[9] Mátyus P, Éliás O, Tapolcsányi P, Polonka-Bálint Á, Halász-Dajka B (2006). Synthesis.

[10] Földi Á A, Ludányi K, Bényei A C, Mátyus P (2010). Synlett.

[11] Dunkel P, Túrós D, Bényei A, Ludányi K, Mátyus P (2010). Tetrahedron.

[12] Murarka S, Deb I, Zhang C, Seidel D (2009). J Am Chem Soc.

[13] Kang Y K, Kim S M, Kim D Y (2010). J Am Chem Soc.

[14] Cao W, Liu X, Wang W, Lin L, Feng X (2011). Org Lett.

[15] Zhou G, Liu F, Zhang J (2011). Chem – Eur J.

[16] Chen L, Zhang L, Lv J, Cheng J-P, Luo S (2012). Chem – Eur J.

[17] Mori K, Ehara K, Kurihara K, Akiyama T (2011). J Am Chem Soc.

[18] Litkey G, Patonay T (1983). Acta Chim Hung.

[19] Zhu J, Ye Y, Ning M, Mándi A, Feng Y, Zou Q, Kurtán T, Leng Y, Shen J (2013). ChemMedChem.

[20] Gulyás-Fekete G, Murillo E, Kurtán T, Papp T, Illyés T-Z, Drahos L, Visy J, Agócs A, Turcsi E, Deli J (2013). J Nat Prod.

[21] Gao H, Liu W, Zhu T, Mo X, Mándi A, Kurtán T, Li J, Ai J, Gu Q, Li D (2012). Org Biomol Chem.

[22] Jiang C-S, Guo X-J, Gong J-X, Zhu T-T, Zhang H-Y, Guo Y-W (2012). Bioorg Med Chem Lett.

[23] Crispo J A G, Piché M, Ansell D R, Eibl J K, Tai I T, Kumar A, Ross G M, Tai T C (2010). Biochem Biophys Res Commun.

[24] Bastianetto S, Yao Z-X, Papadopoulos V, Quirion R (2006). Eur J Neurosci.

[25] (2012). MacroModel.

[26] (2010). Gaussian 09.

[27] Stephens P J, Harada N (2010). Chirality.

[28] (2009). MOLEKEL.

