# Supplementary material for: Preparation of neuroprotective condensed 1,4-benzoxazepines by regio- and diastereoselective domino Knoevenagel–[1,5]-hydride shift cyclization reaction
Source: Beilstein J Org Chem. 2014 Nov 6;10:2594–602. doi: 10.3762/bjoc.10.272 (PMC4273254; doi:10.3762/bjoc.10.272)
Supplement: File 1 — Spectroscopic data and details of calculations. Structures and populations of the in vacuo and PCM solvent model conformers, cartesian coordinates of 7b–d, 1H, 13C NMR and IR spectra are disclosed in this file. [file Beilstein_J_Org_Chem-10-2594-s001.pdf]

Supporting Information

for

Preparation of neuroprotective condensed 1,4-benzoxazepines by regio- and diastereoselective domino Knoevenagel–[1,5]-hydride shift cyclization reaction

László Tóth[1,2], Yan Fu[3], Hai Yan Zhang[3], Attila Mándi[2], Katalin E. Kövér[4], Tünde-Zita Illyés[2], Attila Kiss-Szikszai[2], Balázs Balogh[1], Tibor Kurtán[2,5], Sándor Antus[*2], and Péter Mátyus[*1]

Address: [1]Department of Organic Chemistry, Semmelweis University, Hőgyes u. 7., 1092 Budapest, Hungary, Fax: +36 1 217-0851, [2]Department of Organic Chemistry, University of Debrecen, Debrecen, P. O. Box 20, 4010 Debrecen, Hungary, [3]CAS Key Laboratory of Receptor Research, Shanghai Institute of Materia Medica, Chinese Academy of Sciences, 555 Zu Chong Zhi Road, Zhang Jiang Hi-Tech Park, Shanghai 201203, PR China, [4]Department of Inorganic and Analytical Chemistry, University of Debrecen, H-4010 Debrecen, Hungary and [5]Porfirin Ltd., Mikszáth K. u. 7. III/3, 4032 Debrecen, Hungary

Email: Sándor Antus - antus.sandor@science.unideb.hu; Péter Mátyus - peter.matyus@szerves.sote.hu

*Corresponding author

Spectroscopic data and details of calculations

Table of Contents

Figure S1. Computed structures and population of low-energy conformers of *trans*-(2*S*,15a*S*)-**7b** 3

Figure S2 Computed structures and population of low-energy conformers of *cis*-(2*R*,15a*S*)-**7b** S3

Table S1 Cartesian coordinates of the low-energy DFT-optimized MMFF conformers of *trans*-(2*S*,15a*S*)-**7d**, model compound of **7a** S4

Table S2 Cartesian coordinates of the low-energy DFT-optimized MMFF conformers of *trans*-(2*S*,15a*S*)-**7b** S5

Table S3 Cartesian coordinates of the low-energy DFT-optimized MMFF conformers of *cis*-(2*R*,15a*S*)-**7d** S7

Table S4 Cartesian coordinates of the low-energy DFT-optimized MMFF conformers of *cis*-(2*R*,15a*S*)-**7b** S8

Figure S3. ^{1}H-NMR spectra of (rac)-**9**.. ..... S10

Figure S4. ^{13}C-NMR spectra of (*rac*)-**9**. S11

Figure S5**.** IR spectra of (*rac*)-**9** recorded as KBr disc. S12

Figure S6.^{1}H-NMR spectra of (*rac*)-**10**.. ..... S13

Figure S7. ^{13}C-NMR spectra of (*rac*)-**10**. S14

Figure S8. IR spectra of (*rac*)-**10** recorded as KBr disc. S15

Figure S9.^{1}H-NMR spectra of (*rac*)-**5**.. ..... S16

Figure S10. ^{13}C-NMR spectra of (*rac*)-**5**. S17

Figure S11. IR spectra of (*rac*)-**5** recorded as KBr disc. S18

Figure S12.^{1}H-NMR spectra of (*rac*)-**7a**.. ..... S19

Figure S13. ^{13}C-NMR spectra of (*rac*)-**7a**. S20

Figure S14. IR spectra of (*rac*)-**7a** recorded as KBr disc S21

Figure S15.^{1}H-NMR spectra of (*rac*)-**7b**. S22

Figure S16. ^{13}C-NMR spectra of (*rac*)-**7b**. S23

Figure S17**.** IR spectra of (*rac*)-**7b** recorded as KBr disc. S24

Figure S18.^{1}H-NMR spectra of (*rac*)-**7c**. S25

Figure S19. ^{13}C-NMR spectra of (*rac*)-**7c**. S26

Figure S20. IR spectra of (*rac*)-**7c** recorded as KBr disc. S27

Figures

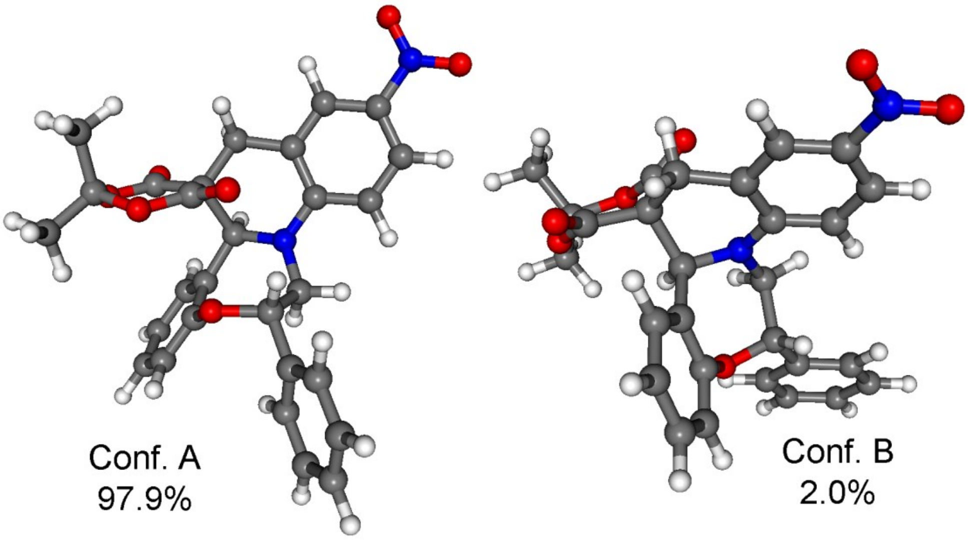

Figure S1. Low-energy conformers (> 1%) of *trans*-(2*S*,15a*S*)-**7b** optimized at B3LYP/6-31G(d) *in vacuo*. Boltzmann-weights for the same conformers in the B3LYP/TZVP PCM/$CHCl_3$ calculations are 95.8% and 3.6%, respectively.

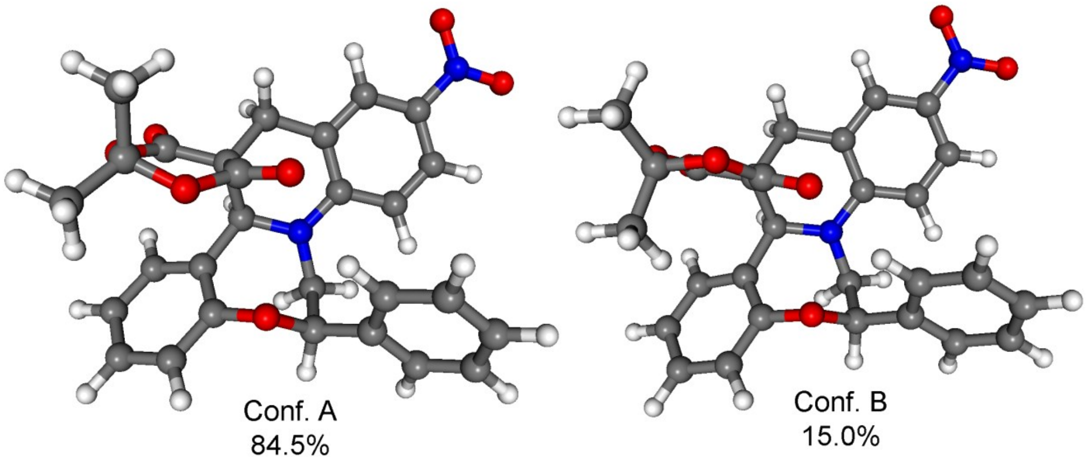

Figure S2. Low-energy conformers (> 1%) of *cis*-(2*R*,15a*S*)-**7b** optimized at B3LYP/6-31G(d) *in vacuo*. Boltzmann-weights for the same conformers in the B3LYP/TZVP PCM/$CHCl_3$ calculations are 82.5% and 14.1%, respectively.

Tables

Table S1. Cartesian coordinates of the low-energy DFT-optimized MMFF conformers of *trans*-(2*S*,15a*S*)-**7d**, model compound of **7a**, calculated at B3LYP/6-31G(d) level of theory *in vacuo*.

**Conformer A**		Standard Orientation (Ångstroms)		
I	Atom	X	Y	Z
1	C	1.877613	-1.936478	-2.305506
2	C	3.235946	-2.181183	-2.508297
3	C	4.177468	-1.600925	-1.657664
4	C	3.759724	-0.764479	-0.623027
5	H	1.143020	-2.405060	-2.954347
6	H	3.553140	-2.825092	-3.323043
7	H	5.237380	-1.791301	-1.801270
8	H	4.469357	-0.293244	0.049089
9	C	0.480525	1.478316	-0.872731
10	C	1.327253	1.512783	0.409322
11	O	2.025182	0.256440	0.643615
12	C	2.398599	-0.518403	-0.427019
13	C	1.433017	-1.119212	-1.257204
14	H	1.124309	1.405059	-1.755443
15	H	-0.022859	2.440012	-0.941635
16	C	-0.057932	-0.972252	-1.016289
17	N	-0.509407	0.407565	-0.890426
18	N	0.768155	-3.880898	0.702833
19	C	-0.173601	-3.309465	-0.132108
20	C	-0.617175	-1.880054	0.186767
21	C	-0.177653	-1.369035	1.553308
22	N	0.779257	-2.119065	2.226588
23	C	1.306858	-3.351488	1.873538
24	O	-0.591066	-3.912411	-1.105896
25	O	-0.642488	-0.365368	2.061528
26	O	2.143386	-3.933207	2.531312
27	C	-2.694114	-0.418384	-0.219461
28	C	-1.864011	0.639208	-0.676231
29	C	-2.454841	1.902893	-0.916865
30	C	-3.805418	2.121260	-0.711439
31	C	-4.601938	1.069722	-0.258516
32	C	-4.049376	-0.184009	-0.013695
33	H	-1.860667	2.723597	-1.299312
34	H	-4.253481	3.087472	-0.907277
35	H	-4.693249	-0.975443	0.353601
36	C	4.013443	4.902816	0.480949
37	C	4.141167	3.938756	-0.521639
38	C	3.282838	2.839883	-0.552646
39	C	2.286805	2.685723	0.423944
40	C	2.170983	3.656616	1.426211
41	C	3.025241	4.759783	1.455442
42	H	4.681854	5.759240	0.502314
43	H	4.909888	4.043293	-1.282680
44	H	3.400203	2.095161	-1.334881
45	H	1.406765	3.544937	2.191941
46	H	2.921075	5.502968	2.241289
47	H	1.125089	-1.728068	3.095999
48	H	1.107413	-4.801060	0.444215
49	H	-0.561443	-1.419700	-1.882054
50	H	0.648432	1.584035	1.262028
51	C	-2.158123	-1.803690	0.063265
52	H	-2.435673	-2.487314	-0.746521
53	H	-2.621269	-2.191827	0.977657
54	N	-6.025064	1.284735	-0.035933
55	O	-6.476350	2.414775	-0.243315
56	O	-6.702371	0.326492	0.347060

B3LYP Energy = -1673.11036861 a.u.; E+ZPVE = -1672.678981 a.u.

Table S2. Cartesian coordinates of the low-energy DFT-optimized MMFF conformers of *trans*-(2*S*,15a*S*)-**7b** calculated at B3LYP/6-31G(d) level of theory *in vacuo*.

**Conformer A**		Standard Orientation (Ångstroms)		
I	Atom	X	Y	Z
1	C	1.862775	-1.540671	-2.568441
2	C	3.219378	-1.768063	-2.799962
3	C	4.165682	-1.277079	-1.900194
4	C	3.754151	-0.545010	-0.786498
5	H	1.125110	-1.944372	-3.255784
6	H	3.530925	-2.331419	-3.674284
7	H	5.224698	-1.454088	-2.066988
8	H	4.467397	-0.139263	-0.076333
9	C	0.478179	1.721045	-0.803473
10	C	1.335412	1.628281	0.469349
11	O	2.028958	0.355685	0.577292
12	C	2.394138	-0.315300	-0.561691
13	C	1.424298	-0.829877	-1.442968
14	H	1.116020	1.732887	-1.693451
15	H	-0.023265	2.685722	-0.774382
16	C	-0.065831	-0.702777	-1.184444
17	N	-0.514425	0.658864	-0.921206
18	O	0.752749	-3.760632	0.209153
19	C	-0.167480	-3.108433	-0.536256
20	C	-0.630136	-1.725943	-0.076365
21	C	-0.193217	-1.340478	1.329520
22	O	0.776636	-2.095538	1.914958
23	C	0.917228	-3.485905	1.612233
24	O	-0.530690	-3.584557	-1.586141
25	O	-0.641466	-0.385853	1.915251
26	C	-0.127151	-4.281266	2.392822
27	C	-2.703371	-0.228966	-0.351787
28	C	-1.868173	0.869635	-0.689238
29	C	-2.456712	2.153209	-0.795551
30	C	-3.808508	2.350464	-0.578431
31	C	-4.610208	1.257941	-0.247887
32	C	-4.059785	-0.015471	-0.133344
33	H	-1.859149	3.009799	-1.081682
34	H	-4.253732	3.333110	-0.672528
35	H	-4.707225	-0.840349	0.142909
36	C	4.034258	4.991408	0.824432
37	C	3.048694	4.767843	1.786449
38	C	2.190519	3.673904	1.665365
39	C	2.299598	2.792802	0.582636
40	C	3.293086	3.027556	-0.380356
41	C	4.155489	4.116762	-0.257742
42	H	4.705668	5.840747	0.917299
43	H	2.949786	5.440503	2.634130
44	H	1.429200	3.497613	2.421754
45	H	3.405404	2.352798	-1.224326
46	H	4.922293	4.283959	-1.009551
47	H	-0.572403	-1.059938	-2.088792
48	H	0.666325	1.622755	1.332511
49	C	2.350591	-3.855588	1.949989
50	H	-0.003558	-4.106276	3.464952
51	H	-1.139990	-3.975699	2.107635
52	H	-0.017505	-5.349248	2.185733
53	H	2.540484	-3.669016	3.010200
54	H	2.523766	-4.912718	1.732069
55	H	3.033968	-3.247932	1.351623
56	C	-2.170077	-1.637351	-0.206621
57	H	-2.442870	-2.234610	-1.083437
58	H	-2.644581	-2.113833	0.659482
59	N	-6.034488	1.449919	-0.016742
60	O	-6.715789	0.457709	0.258524
61	O	-6.484317	2.595855	-0.109336

B3LYP Energy = -1717.41808982 a.u.; E+ZPVE = -1716.938660 a.u.

Conformer B		Standard Orientation (Ångstroms)		
I	Atom	X	Y	Z
1	C	0.757476	0.793089	2.760935
2	C	0.425583	0.503770	4.087268
3	C	-0.851806	0.052817	4.410191
4	C	-1.797304	-0.119031	3.399154
5	H	1.746886	1.175265	2.551161
6	H	1.172009	0.640591	4.864118
7	H	-1.114280	-0.168369	5.440918
8	H	-2.803176	-0.467368	3.611985
9	C	-1.401047	-0.684533	-0.965442
10	C	-2.305412	-1.105728	0.212170
11	O	-2.443810	0.011287	1.116996
12	C	-1.458360	0.161173	2.077599
13	C	-0.175800	0.636207	1.730268
14	H	-1.870449	0.178127	-1.454064
15	H	-1.347786	-1.477944	-1.713867
16	C	0.042862	0.923994	0.240297
17	N	-0.056985	-0.301507	-0.563403
18	O	0.219604	2.973619	-1.943994
19	C	1.133246	1.985128	-1.699330
20	C	1.357443	1.667674	-0.209439
21	C	1.505270	2.990960	0.550428
22	O	0.585062	3.941346	0.219248
23	C	0.196027	4.171929	-1.148160
24	O	1.624260	1.372513	-2.607651
25	O	2.298186	3.197942	1.431937
26	C	1.177823	5.169329	-1.758821
27	C	2.308420	-0.710478	-0.256833
28	C	1.002536	-1.199039	-0.525384
29	C	0.828689	-2.579917	-0.772764
30	C	1.902187	-3.455043	-0.749358
31	C	3.174126	-2.959804	-0.467340
32	C	3.373546	-1.601184	-0.226828
33	H	-0.158307	-2.980606	-0.968691
34	H	1.767931	-4.514179	-0.930899
35	H	4.380076	-1.247944	-0.031385
36	C	-6.179652	-2.291693	-1.279362
37	C	-5.935884	-0.955266	-0.956526
38	C	-4.692207	-0.567260	-0.457544
39	C	-3.679201	-1.515713	-0.269498
40	C	-3.932549	-2.855026	-0.589485
41	C	-5.174344	-3.242150	-1.094213
42	H	-7.149392	-2.591754	-1.666917
43	H	-6.717310	-0.211993	-1.090854
44	H	-4.508615	0.468367	-0.190010
45	H	-3.157553	-3.603151	-0.434048
46	H	-5.358177	-4.286050	-1.333346
47	H	-0.798632	1.546116	-0.074599
48	H	-1.835297	-1.937794	0.751706
49	C	-1.240657	4.661582	-1.121482
50	H	1.173482	6.098424	-1.182654
51	H	2.193476	4.759878	-1.750871
52	H	0.897844	5.382037	-2.794036
53	H	-1.306645	5.583822	-0.538419
54	H	-1.584012	4.855919	-2.140978
55	H	-1.887456	3.907558	-0.664991
56	C	2.589346	0.763748	-0.041711
57	H	3.353967	1.085422	-0.756788
58	H	3.015822	0.925553	0.950943
59	N	4.309780	-3.870845	-0.425512
60	O	5.419553	-3.400530	-0.159366
61	O	4.100130	-5.065009	-0.657637

B3LYP Energy = -1717.41427030 a.u.; E+ZPVE = -1716.934998 a.u.

Table S3. Cartesian coordinates of the low-energy DFT-optimized MMFF conformers of *cis*-(2*R*,15a*S*)-**7d**, model compound of **7a**, calculated at B3LYP/6-31G(d) level of theory *in vacuo*.

**Conformer A**		Standard Orientation (Ångstroms)		
I	Atom	X	Y	Z
1	C	3.432189	-0.394337	-1.833627
2	C	4.661339	0.264956	-1.850140
3	C	4.807022	1.479960	-1.180527
4	C	3.714154	2.040514	-0.520622
5	H	3.325285	-1.341882	-2.354280
6	H	5.498532	-0.170025	-2.387906
7	H	5.761177	1.999042	-1.184324
8	H	3.787144	2.995291	-0.009181
9	C	-0.029739	1.445198	-1.740367
10	C	0.438735	2.529093	-0.730447
11	O	1.430934	1.971681	0.153553
12	C	2.487493	1.375508	-0.503802
13	C	2.330419	0.129866	-1.141009
14	H	0.702930	1.372879	-2.550493
15	H	-0.961173	1.759573	-2.208635
16	C	1.042491	-0.685694	-1.081320
17	N	-0.175006	0.128939	-1.132634
18	N	3.261636	-2.442646	0.910401
19	C	2.237828	-2.602761	-0.003966
20	C	1.007671	-1.699032	0.151337
21	C	0.950112	-0.959918	1.486619
22	N	2.146851	-0.843808	2.186603
23	C	3.317621	-1.562482	1.989084
24	O	2.336428	-3.407936	-0.913200
25	O	-0.069864	-0.474744	1.934236
26	O	4.292849	-1.459481	2.701292
27	C	-1.493714	-1.767195	-0.367216
28	C	-1.414222	-0.444502	-0.878893
29	C	-2.623597	0.262455	-1.087565
30	C	-3.854393	-0.328792	-0.863436
31	C	-3.908531	-1.643146	-0.401937
32	C	-2.735205	-2.345164	-0.137641
33	H	-2.612422	1.299497	-1.394226
34	H	-4.773723	0.218240	-1.031556
35	H	-2.805909	-3.352526	0.257262
36	C	-2.804765	4.284800	1.515385
37	C	-2.116870	3.189053	2.038637
38	C	-1.056706	2.613545	1.335596
39	C	-0.672455	3.137868	0.096422
40	C	-1.359643	4.245109	-0.419951
41	C	-2.423709	4.813100	0.280525
42	H	-3.629878	4.727120	2.067089
43	H	-2.408040	2.772285	2.999022
44	H	-0.534722	1.757572	1.745818
45	H	-1.055928	4.672877	-1.373894
46	H	-2.945016	5.672728	-0.132111
47	H	2.119084	-0.282533	3.031336
48	H	4.095891	-2.999576	0.759745
49	H	1.058759	-1.368187	-1.943981
50	H	0.909640	3.328796	-1.322968
51	C	-0.255318	-2.568014	-0.045262
52	H	-0.024241	-3.272719	-0.851246
53	H	-0.432078	-3.166952	0.855324
54	N	-5.195788	-2.277205	-0.166964
55	O	-5.201677	-3.440338	0.248579
56	O	-6.214723	-1.618983	-0.397947

B3LYP Energy = -1673.10783505 a.u.; E+ZPVE = -1672.676362 a.u.

Table S4. Cartesian coordinates of the low-energy DFT-optimized MMFF conformers of *cis*-(2*R*,15a*S*)-**7b** calculated at B3LYP/6-31G(d) level of theory *in vacuo*.

**Conformer A**		Standard Orientation (Ångstroms)		
I	Atom	X	Y	Z
1	C	3.285862	0.226919	-2.018875
2	C	4.445873	1.000718	-1.981320
3	C	4.483387	2.157981	-1.203328
4	C	3.350284	2.546246	-0.489105
5	H	3.266522	-0.676217	-2.621474
6	H	5.312725	0.698285	-2.561204
7	H	5.381704	2.767990	-1.166660
8	H	3.336498	3.453474	0.106981
9	C	-0.350026	1.692372	-1.711284
10	C	0.030357	2.722566	-0.612707
11	O	1.100147	2.198945	0.193643
12	C	2.193293	1.766647	-0.526212
13	C	2.148137	0.574383	-1.274941
14	H	0.368209	1.767408	-2.533863
15	H	-1.319930	1.946187	-2.137750
16	C	0.948167	-0.369824	-1.284931
17	N	-0.344653	0.320089	-1.222743
18	O	3.389284	-2.159082	0.374937
19	C	2.348874	-2.270291	-0.475866
20	C	1.051079	-1.510338	-0.172383
21	C	0.999429	-0.907983	1.225607
22	O	2.200913	-0.696158	1.838202
23	C	3.252342	-1.656610	1.719842
24	O	2.483090	-2.908810	-1.493629
25	O	-0.016714	-0.549176	1.761561
26	C	4.544319	-0.924379	2.034748
27	C	-1.456812	-1.748825	-0.585682
28	C	-1.514050	-0.380414	-0.962391
29	C	-2.786699	0.238256	-1.031564
30	C	-3.950065	-0.474796	-0.802453
31	C	-3.871183	-1.827803	-0.474865
32	C	-2.631225	-2.449577	-0.347773
33	H	-2.875248	1.298704	-1.224305
34	H	-4.918343	0.006539	-0.861377
35	H	-2.597452	-3.492772	-0.054019
36	C	-3.313094	3.859951	1.870793
37	C	-2.437961	2.863474	2.304900
38	C	-1.342578	2.489410	1.523813
39	C	-1.113959	3.118150	0.295051
40	C	-1.988478	4.127287	-0.131568
41	C	-3.085935	4.493545	0.647330
42	H	-4.164582	4.145073	2.482846
43	H	-2.607945	2.367104	3.256471
44	H	-0.671847	1.710067	1.865010
45	H	-1.806868	4.636550	-1.076709
46	H	-3.754395	5.278874	0.304499
47	H	1.005452	-0.947444	-2.218871
48	H	0.391688	3.622652	-1.134808
49	C	2.965769	-2.838247	2.643078
50	H	5.390976	-1.610585	1.949325
51	H	4.504229	-0.526299	3.052115
52	H	4.677363	-0.098894	1.331149
53	H	2.882796	-2.491741	3.676715
54	H	2.025016	-3.326096	2.365031
55	H	3.771449	-3.573941	2.571620
56	C	-0.139195	-2.464528	-0.419935
57	H	0.106076	-3.044677	-1.315820
58	H	-0.215132	-3.180691	0.406658
59	N	-5.085883	-2.588258	-0.233483
60	O	-4.973410	-3.780851	0.069026
61	O	-6.167145	-2.002129	-0.345722

B3LYP Energy = -1717.41661743 a.u.; E+ZPVE = -1716.937000 a.u.

Conformer B		Standard Orientation (Ångstroms)		
I	Atom	X	Y	Z
1	C	3.336209	0.721719	-1.817511
2	C	4.404360	1.605039	-1.663468
3	C	4.274075	2.713407	-0.826282
4	C	3.063813	2.943242	-0.175033
5	H	3.446173	-0.137249	-2.471755
6	H	5.330655	1.426728	-2.201573
7	H	5.099802	3.407490	-0.697111
8	H	2.915338	3.811009	0.460221
9	C	-0.467335	1.768702	-1.671394
10	C	-0.246593	2.793681	-0.522075
11	O	0.823939	2.353221	0.327694
12	C	1.994825	2.056054	-0.330151
13	C	2.122593	0.904586	-1.132907
14	H	0.273896	1.952278	-2.455918
15	H	-1.440253	1.934782	-2.135125
16	C	1.032759	-0.159872	-1.293846
17	N	-0.333756	0.385156	-1.239540
18	O	3.434305	-2.369221	0.288765
19	C	2.545609	-2.081232	-0.682406
20	C	1.228627	-1.383992	-0.316596
21	C	1.090359	-1.025809	1.160786
22	O	2.028190	-1.520058	2.017980
23	C	3.366017	-1.831617	1.624687
24	O	2.772626	-2.447784	-1.812353
25	O	0.134251	-0.456126	1.617464
26	C	4.228377	-0.576691	1.699120
27	C	-1.264507	-1.795044	-0.692515
28	C	-1.434240	-0.416190	-0.990612
29	C	-2.753778	0.100829	-0.999186
30	C	-3.848765	-0.710091	-0.759247
31	C	-3.656198	-2.063125	-0.480862
32	C	-2.368741	-2.593434	-0.431107
33	H	-2.932041	1.156568	-1.149524
34	H	-4.852664	-0.304026	-0.767635
35	H	-2.242970	-3.643359	-0.191499
36	C	-3.781399	3.536224	1.842830
37	C	-2.831872	2.615135	2.288004
38	C	-1.677430	2.367948	1.542796
39	C	-1.463645	3.050966	0.340252
40	C	-2.413171	3.984244	-0.097156
41	C	-3.570066	4.222191	0.645083
42	H	-4.678921	3.722050	2.426648
43	H	-2.990503	2.078382	3.219447
44	H	-0.947991	1.643274	1.884104
45	H	-2.244632	4.535714	-1.020783
46	H	-4.297477	4.949630	0.294610
47	H	1.191817	-0.610146	-2.283337
48	H	0.048609	3.741264	-1.000526
49	C	3.833300	-2.945223	2.548189
50	H	5.256357	-0.816373	1.413235
51	H	4.221722	-0.189959	2.722041
52	H	3.850399	0.199084	1.029032
53	H	3.804662	-2.601256	3.585386
54	H	3.178016	-3.813655	2.442438
55	H	4.855657	-3.235847	2.292748
56	C	0.107285	-2.410629	-0.675247
57	H	0.370585	-2.811815	-1.660463
58	H	0.135779	-3.252794	0.025912
59	N	-4.798690	-2.921076	-0.219828
60	O	-4.584290	-4.110433	0.037958
61	O	-5.924983	-2.416651	-0.271244

B3LYP Energy = -1717.41520537 a.u.; E+ZPVE = -1716.935371 a.u.

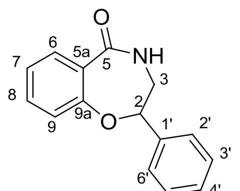

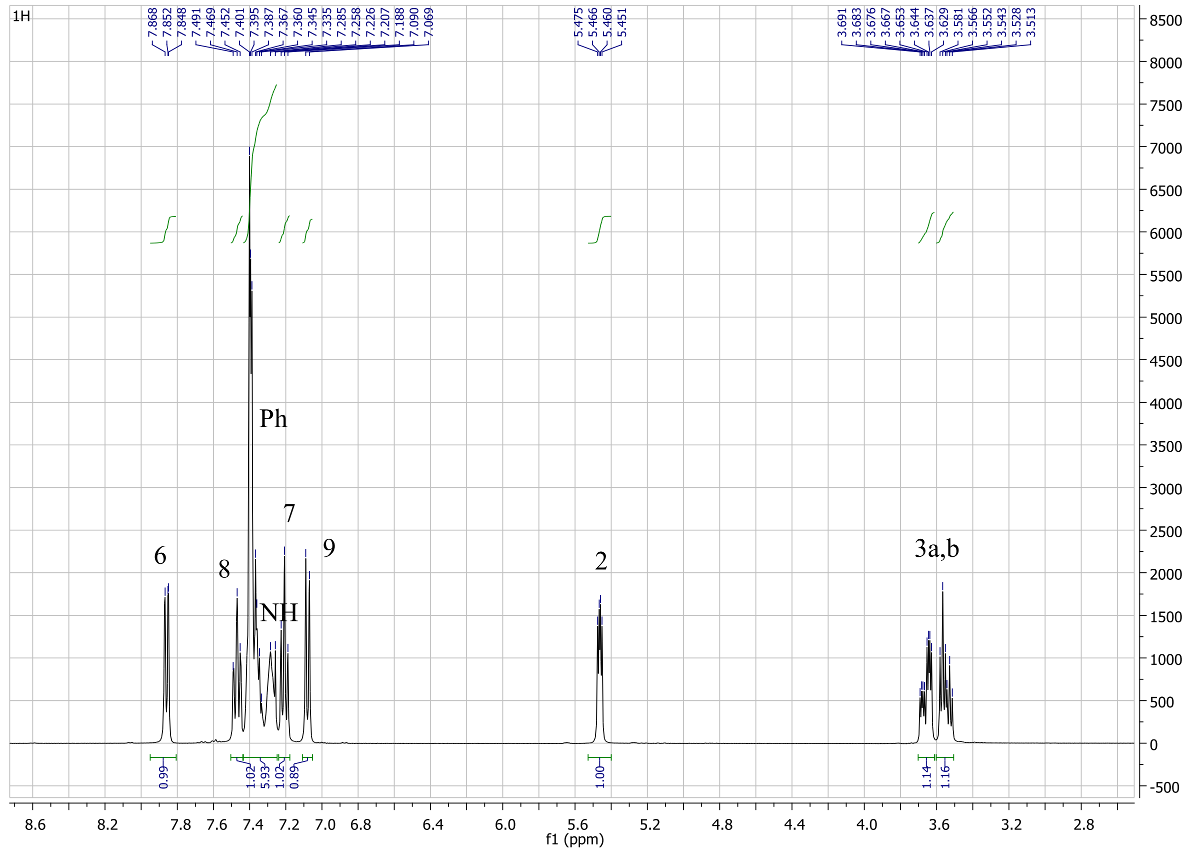

Figure S3. ^{1}H-NMR spectra of (*rac*)-**9** measured in $CDCl_3$ (400 MHz).

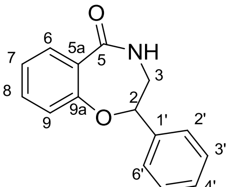

(*rac*)-**9**

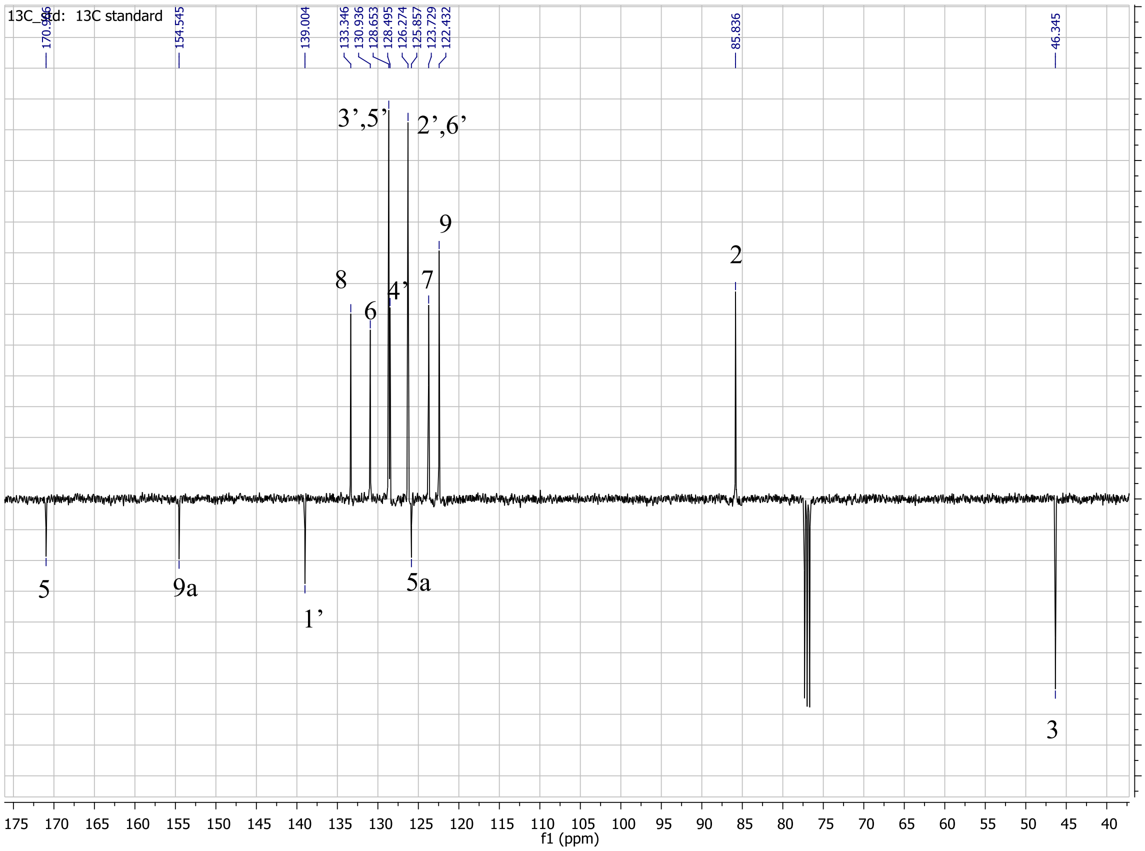

Figure S4. ^{13}C-NMR spectra of (*rac*)-**9** measured in $CDCl_3$ (100 MHz).

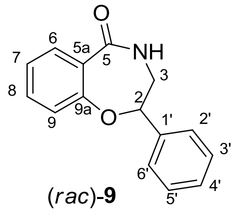

(*rac*)-**9**

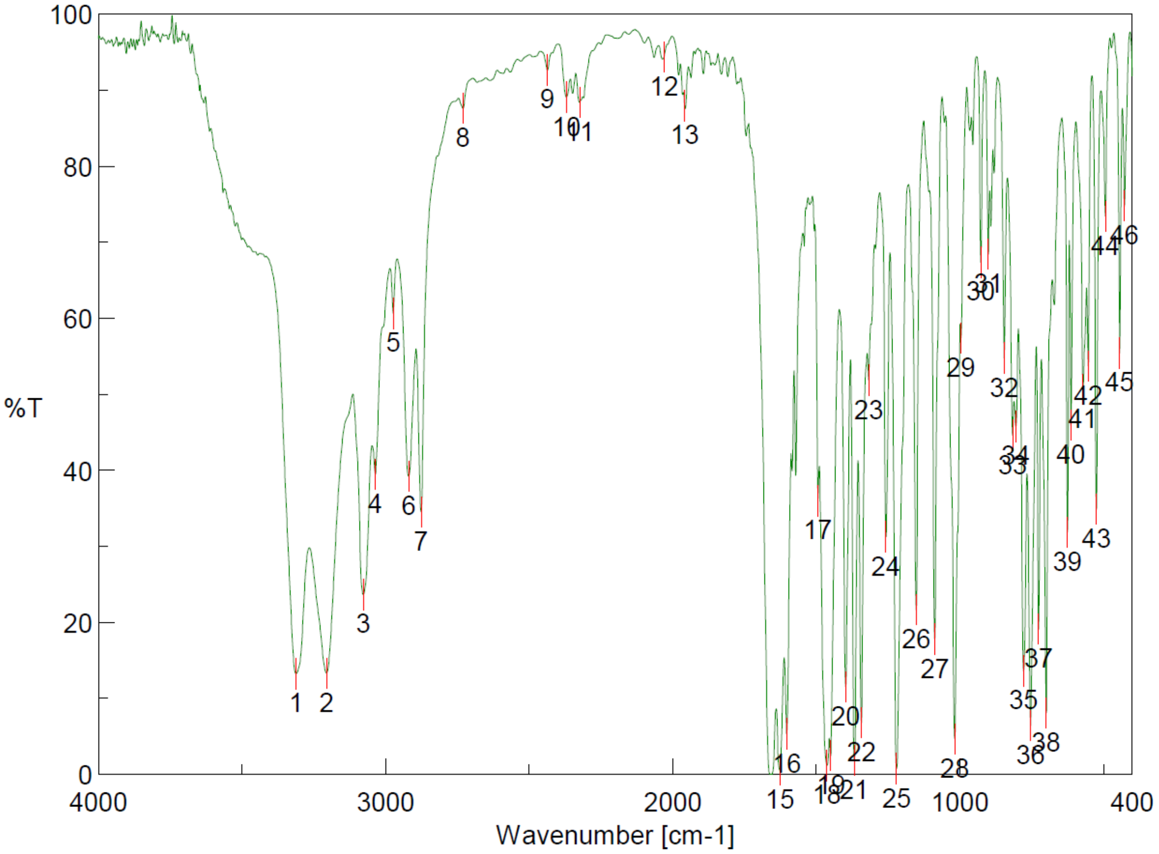

Figure S5. IR spectra of (*rac*)-**9** recorded as KBr disc.

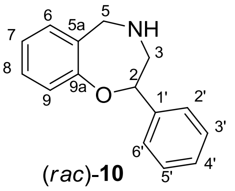

(*rac*)-**10**

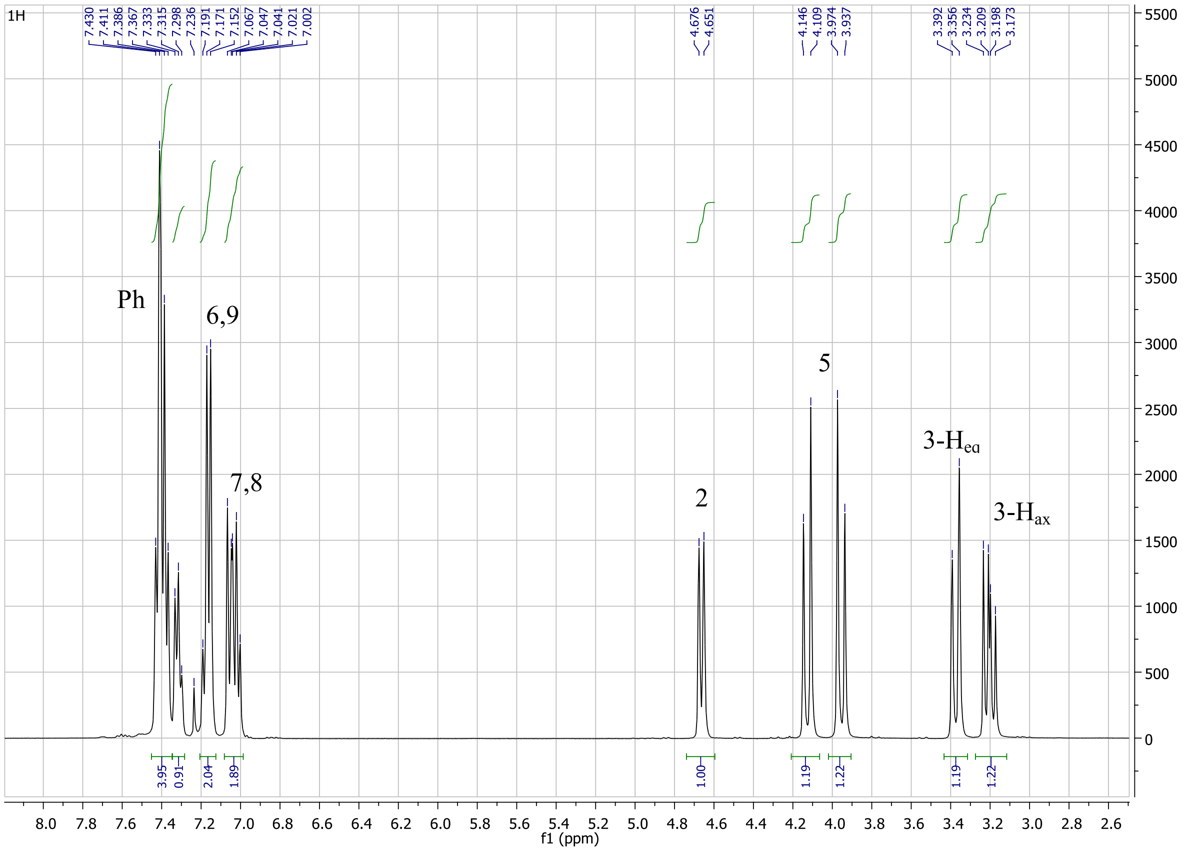

Figure S6. ^{1}H-NMR spectra of (*rac*)-**10** measured in $CDCl_3$ (400 MHz).

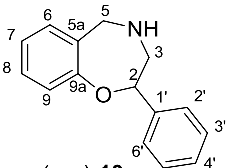

(*rac*)-**10**

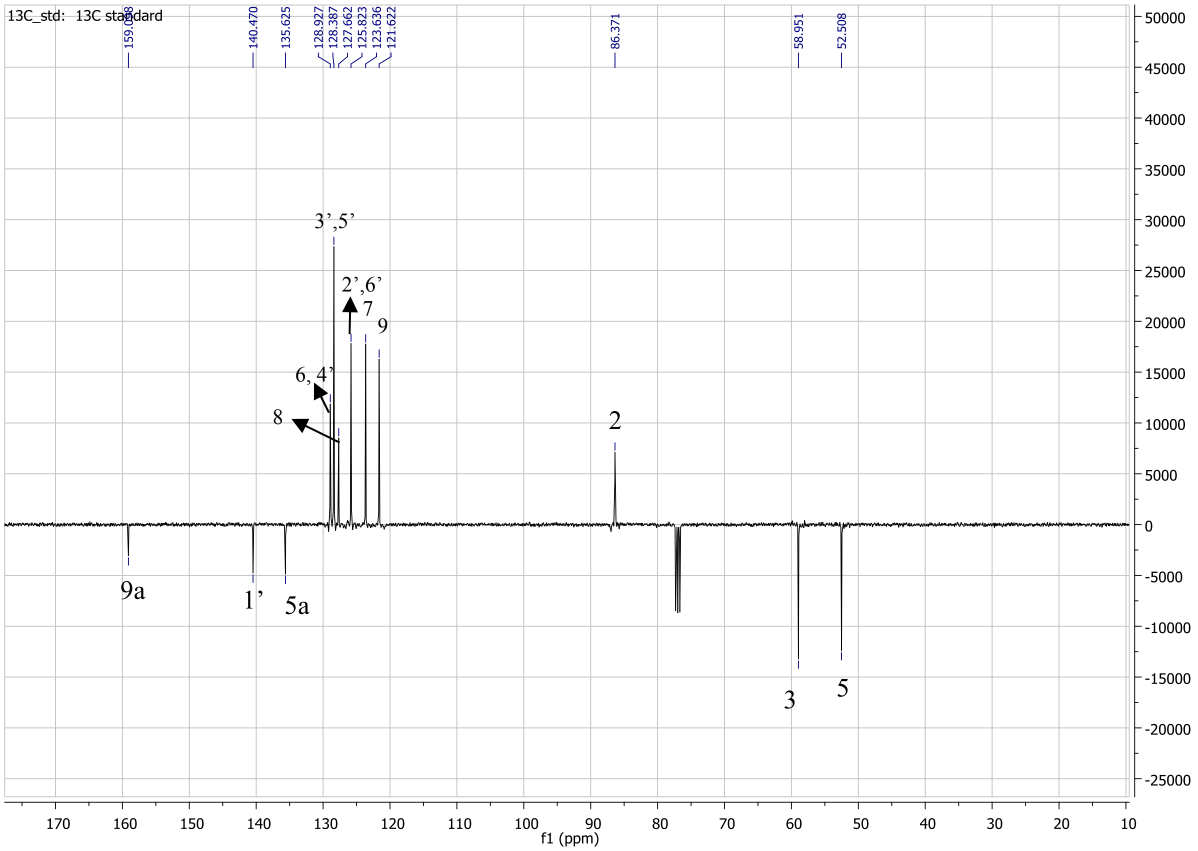

Figure S7. ^{13}C-NMR spectra of (*rac*)-**10** measured in $CDCl_3$ (100 MHz).

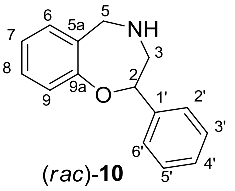

(*rac*)-**10**

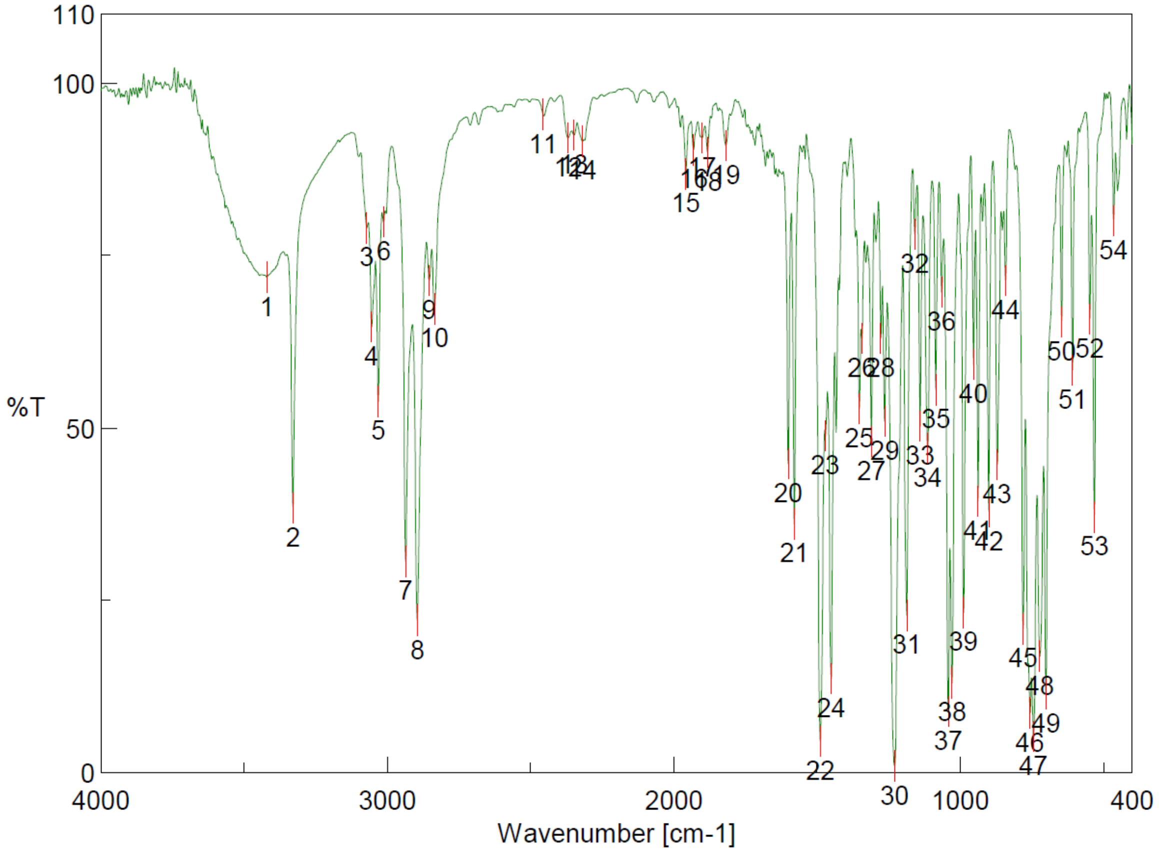

Figure S8. IR spectra of (*rac*)-**10** recorded as KBr disc.

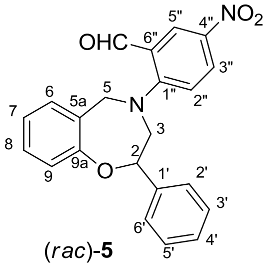

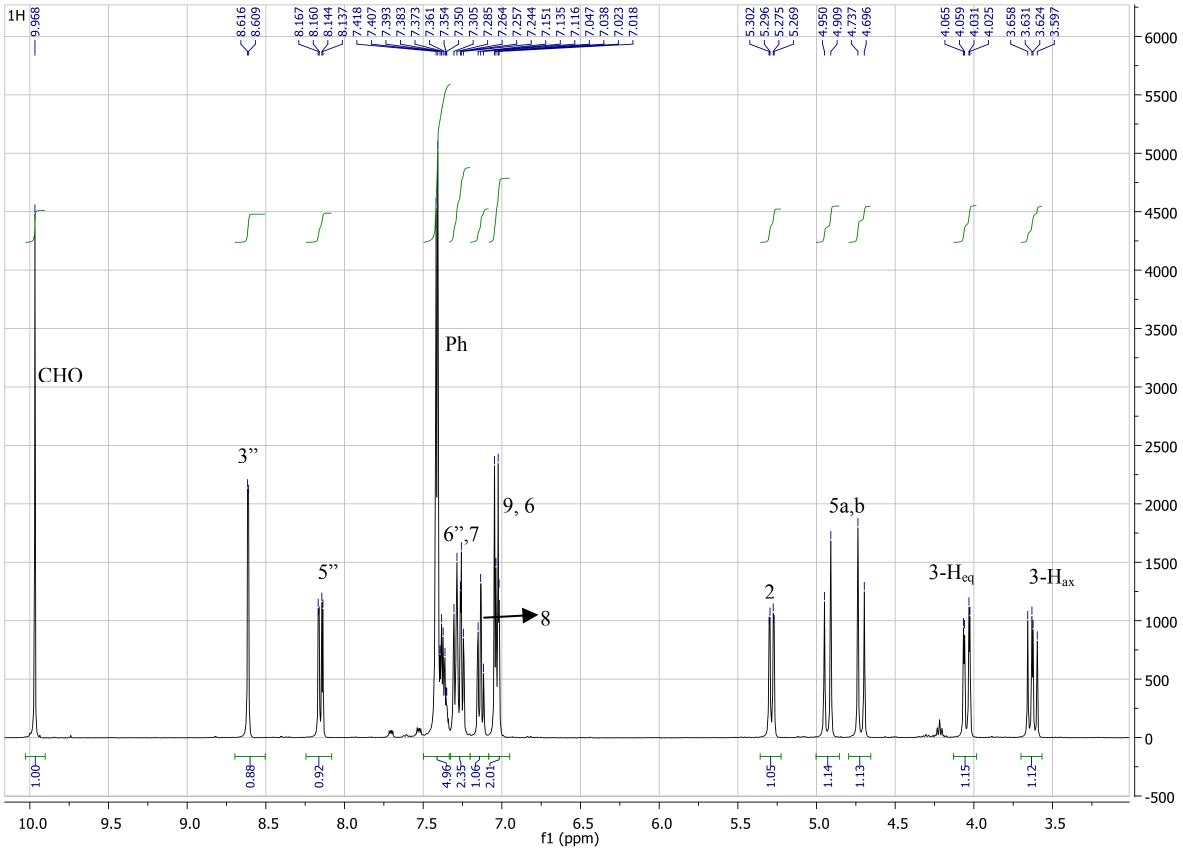

Figure S9. ^{1}H-NMR spectra of (*rac*)-**5** measured in $CDCl_3$ (400 MHz).

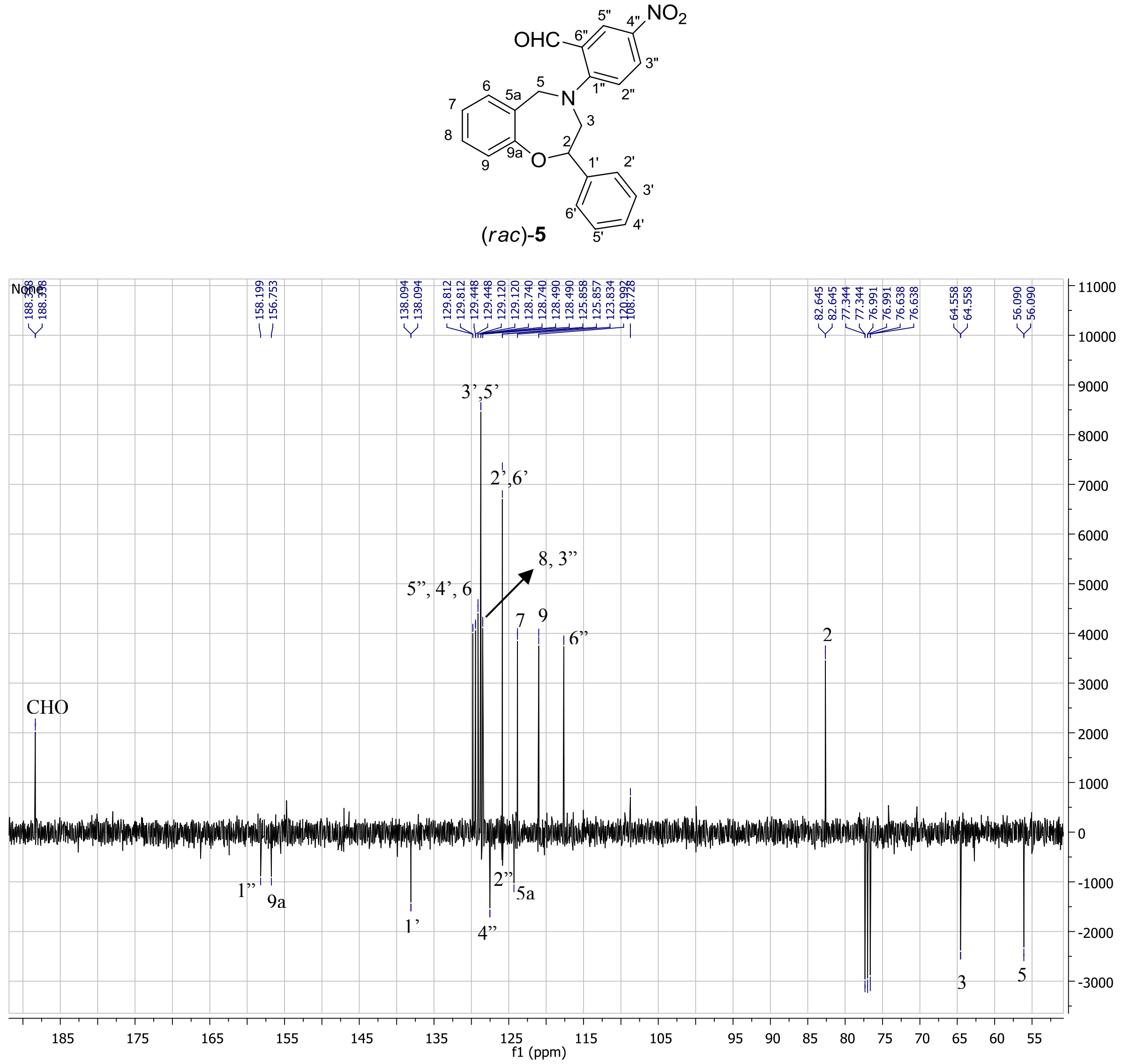

Figure S10. ^{13}C-NMR spectra of (*rac*)-**5** measured in $CDCl_3$ (100 MHz).

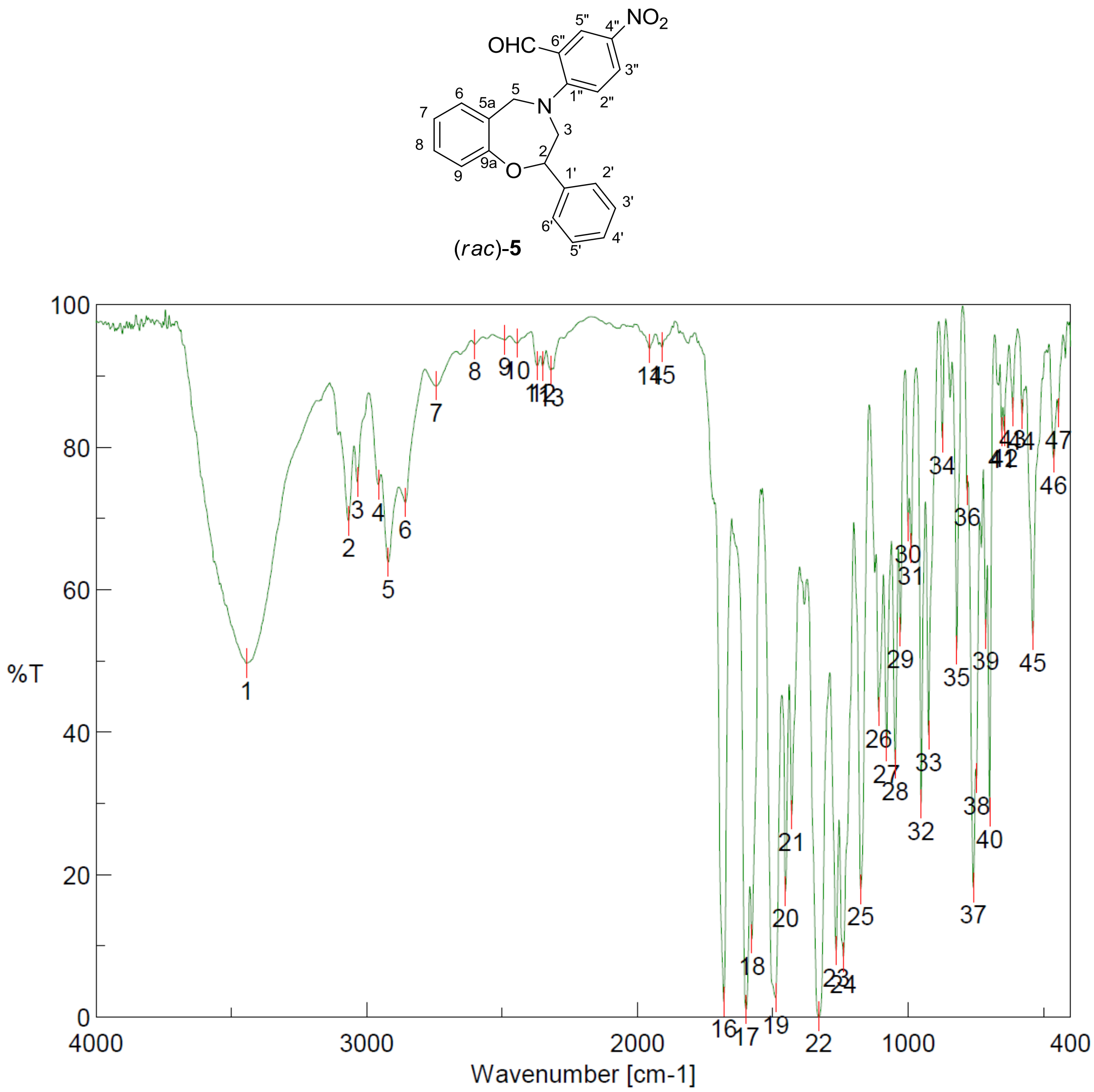

Figure S11. IR spectra of (*rac*)-**5** recorded as KBr disc.

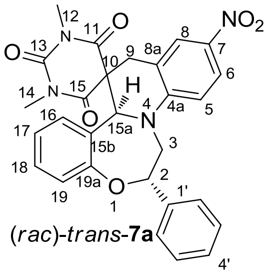

(*rac*)-*trans*-**7a**

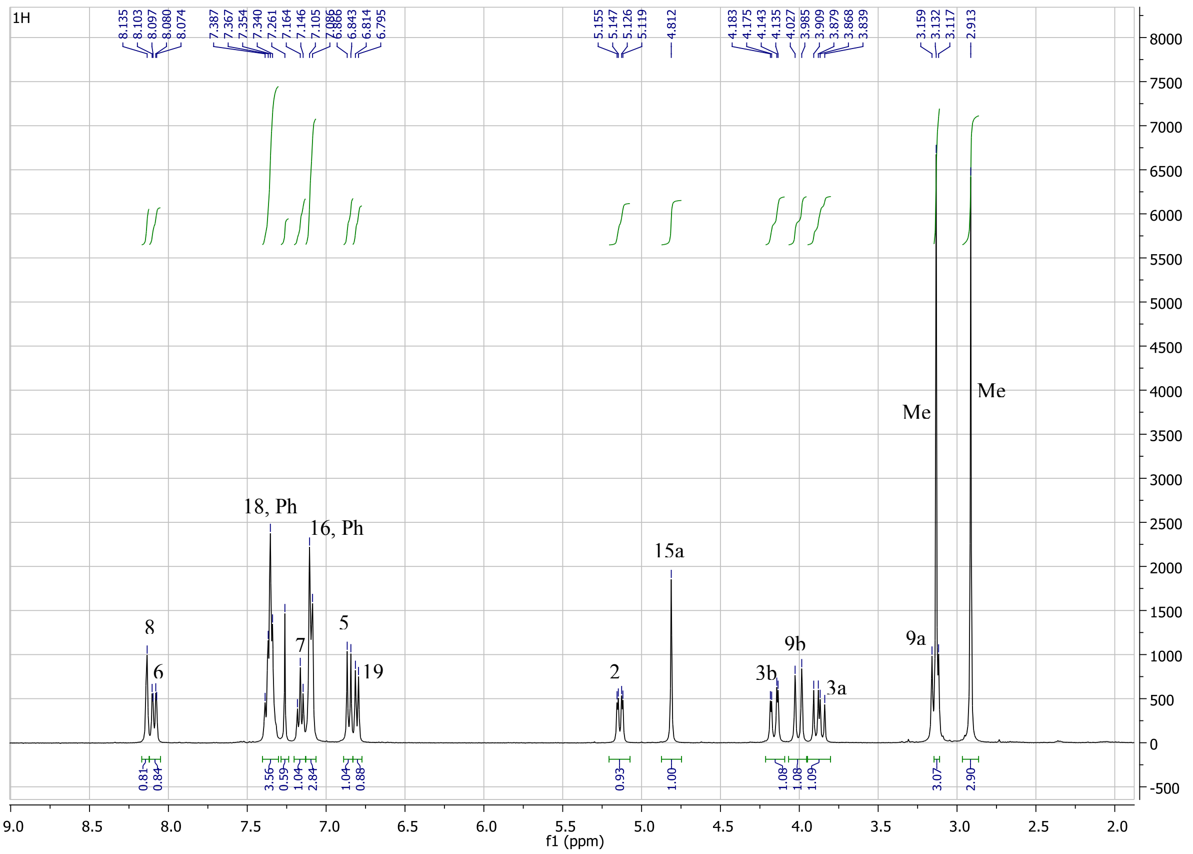

Figure S12.^{1}H-NMR spectra of (*rac*)-**7a** measured in $CDCl_3$ (400 MHz).

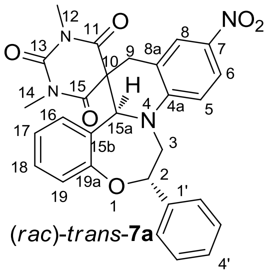

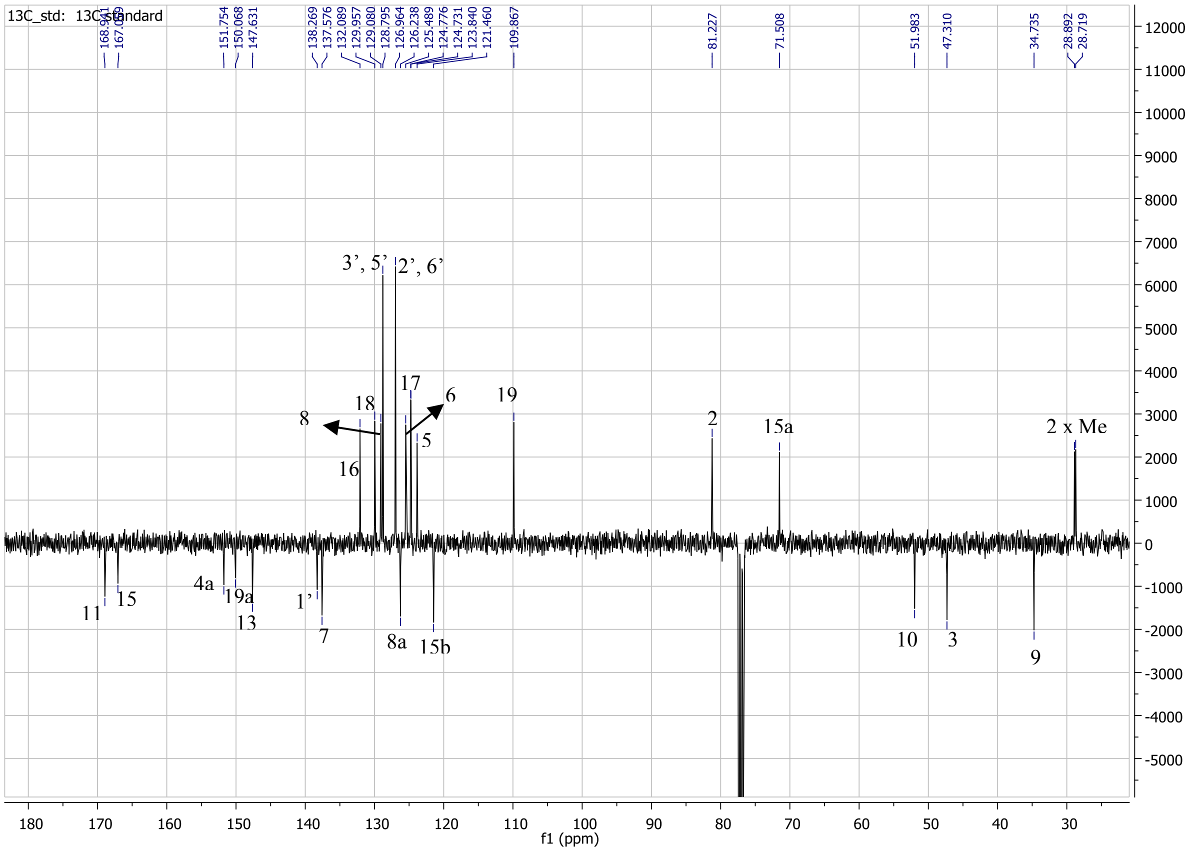

Figure S13. ^{13}C-NMR spectra of (*rac*)-**7a** measured in $CDCl_3$ (100 MHz).

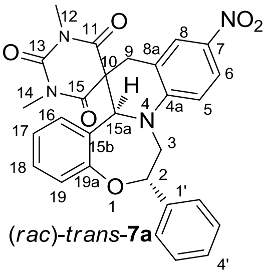

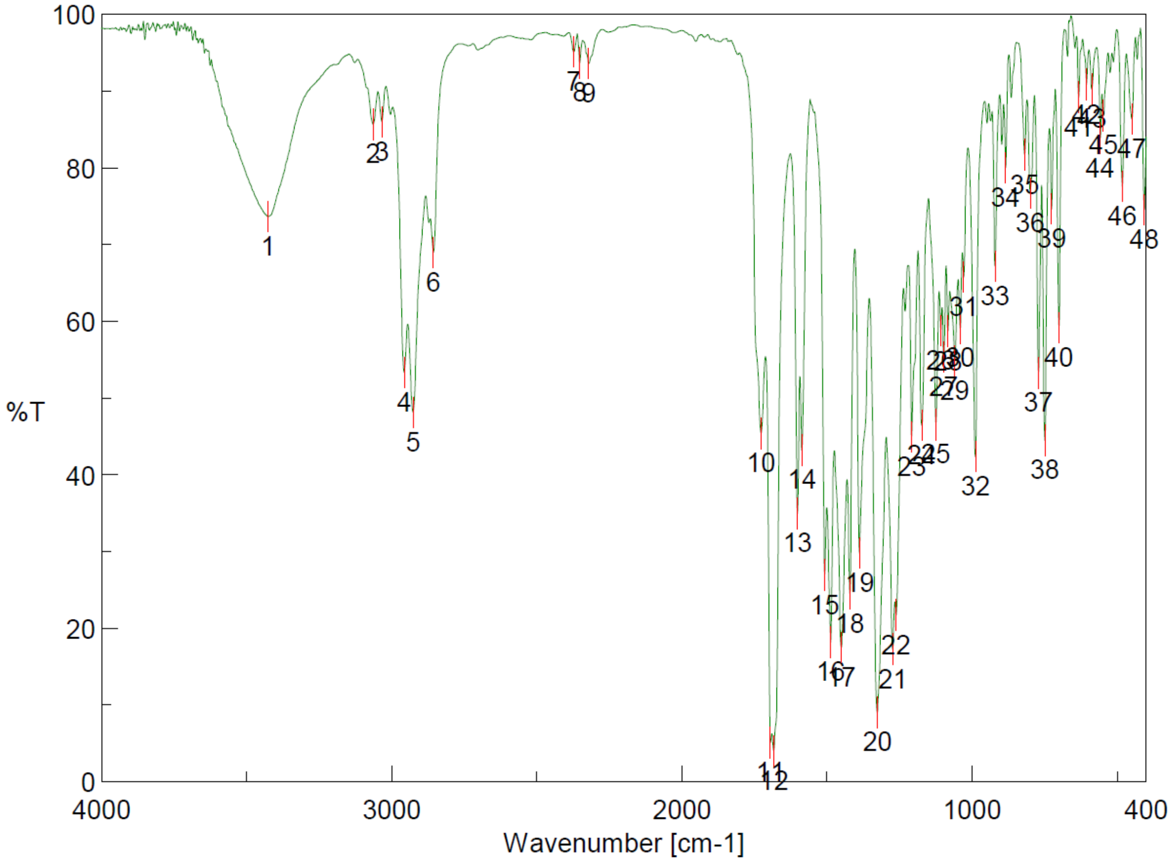

Figure S14. IR spectra of (*rac*)-**7a** recorded as KBr disc.

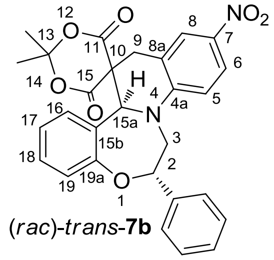

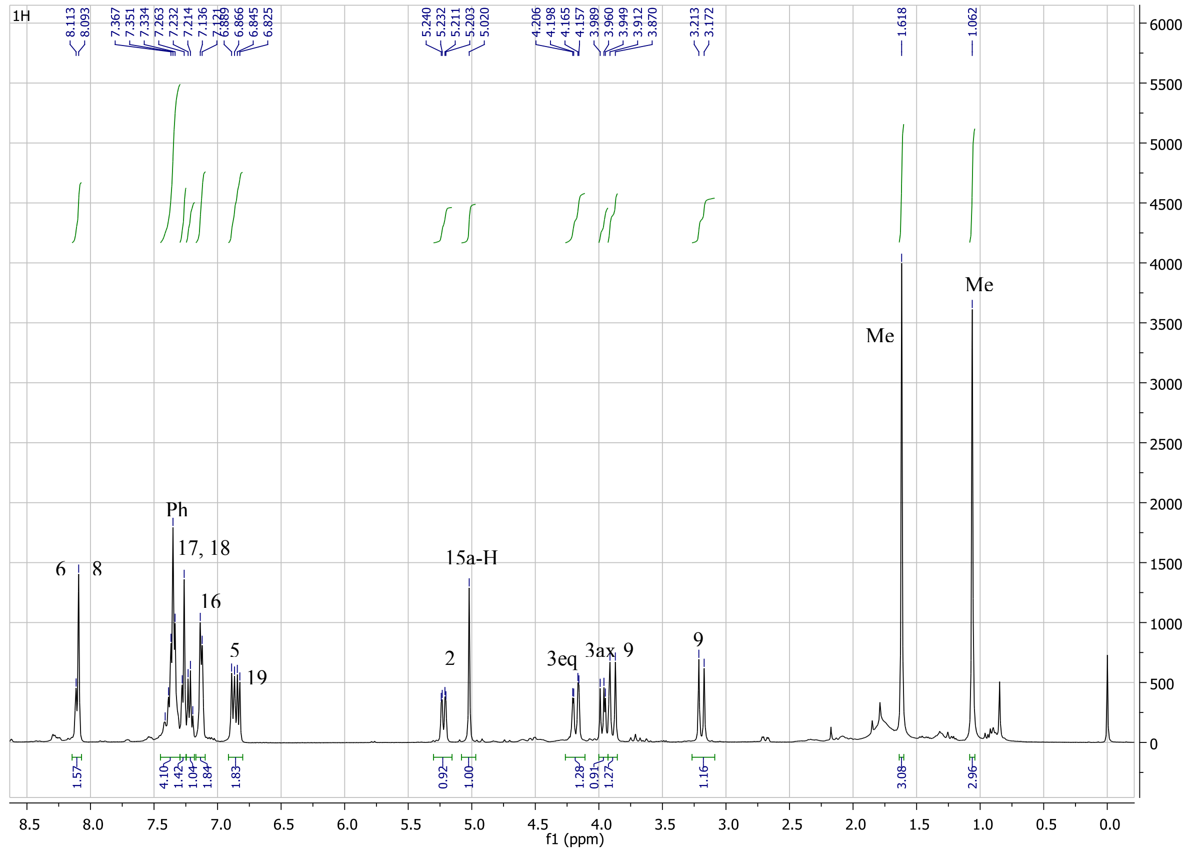

Figure S15.^{1}H-NMR spectra of (*rac*)-**7b** measured in $CDCl_3$ (400 MHz).

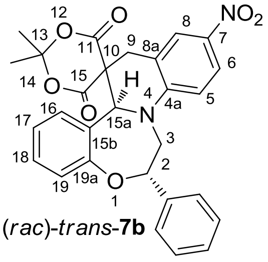

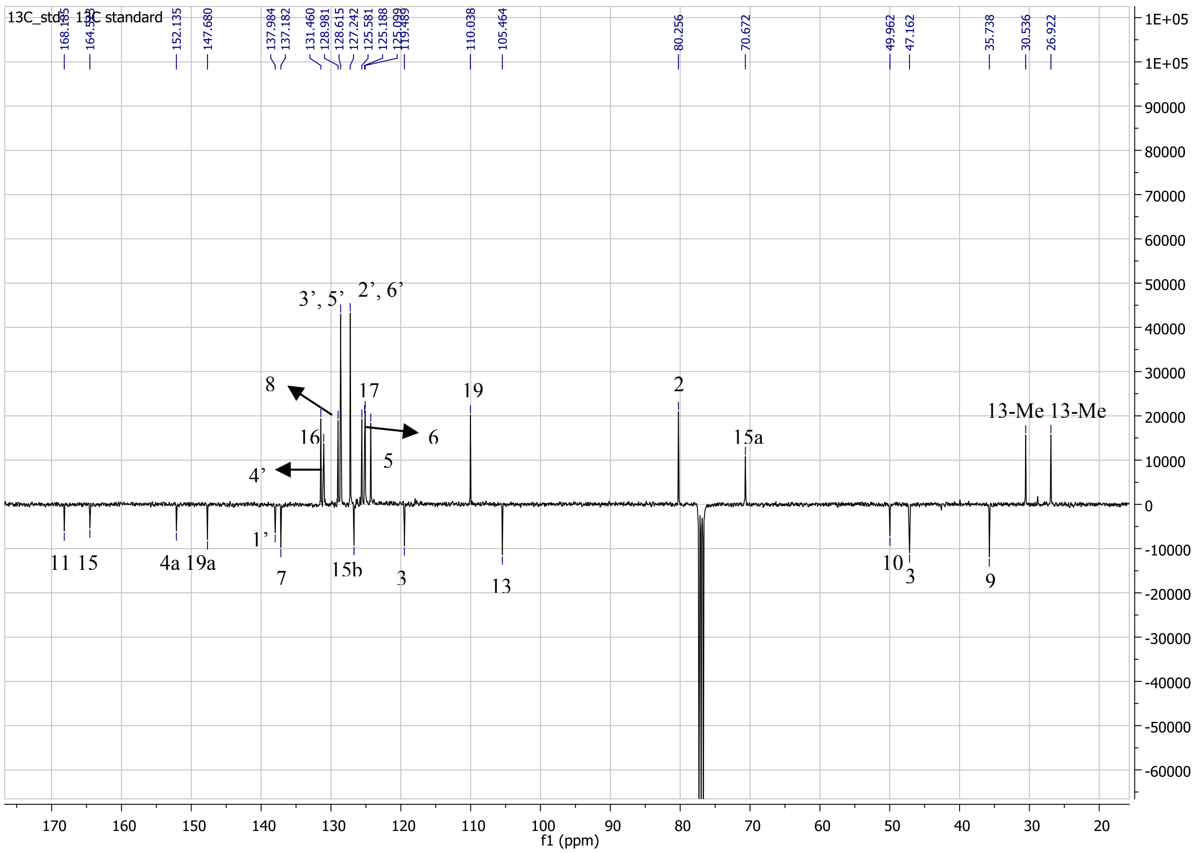

Figure S16.^{13}C-NMR spectra of (*rac*)-**7b** measured in $CDCl_3$ (100 MHz).

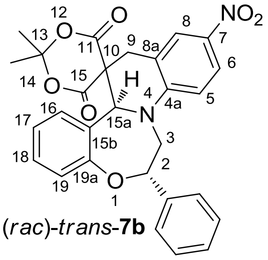

(*rac*)-*trans*-**7b**

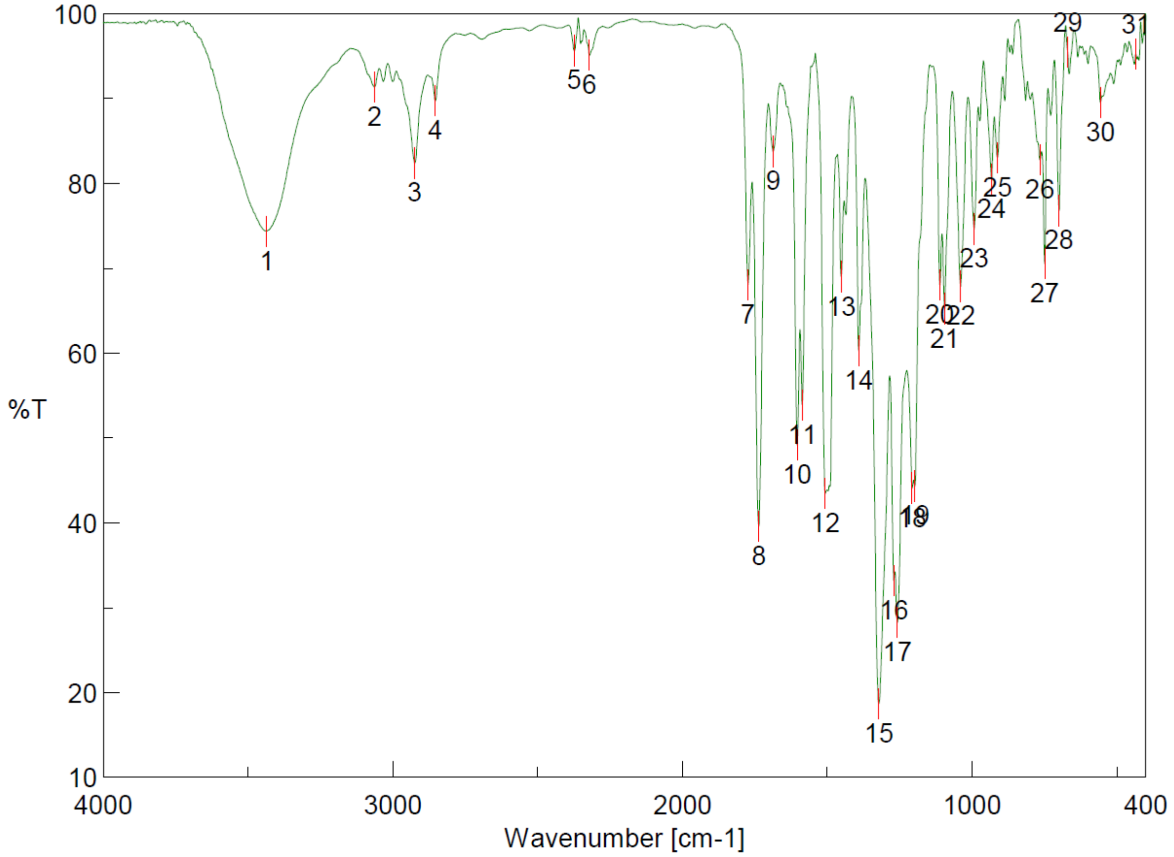

Figure S17. IR spectra of (*rac*)-**7b** recorded as KBr disc.

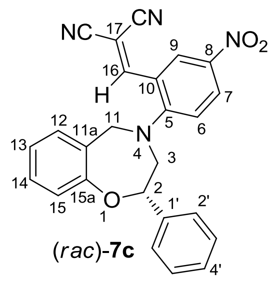

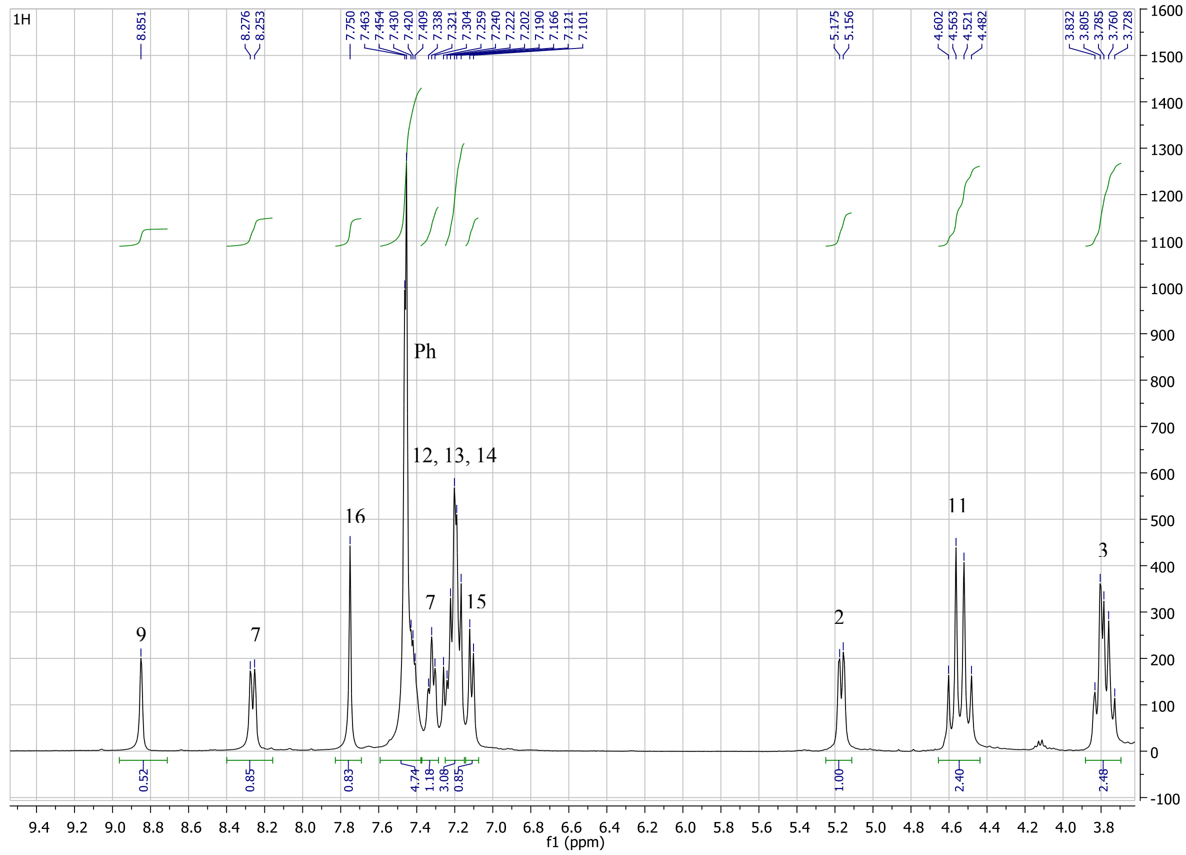

Figure S18. ¹H-NMR spectra of (*rac*)-**7c** measured in $CDCl_3$ 400 MHz.

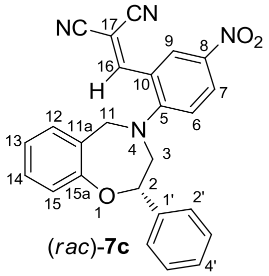

(*rac*)-**7c**

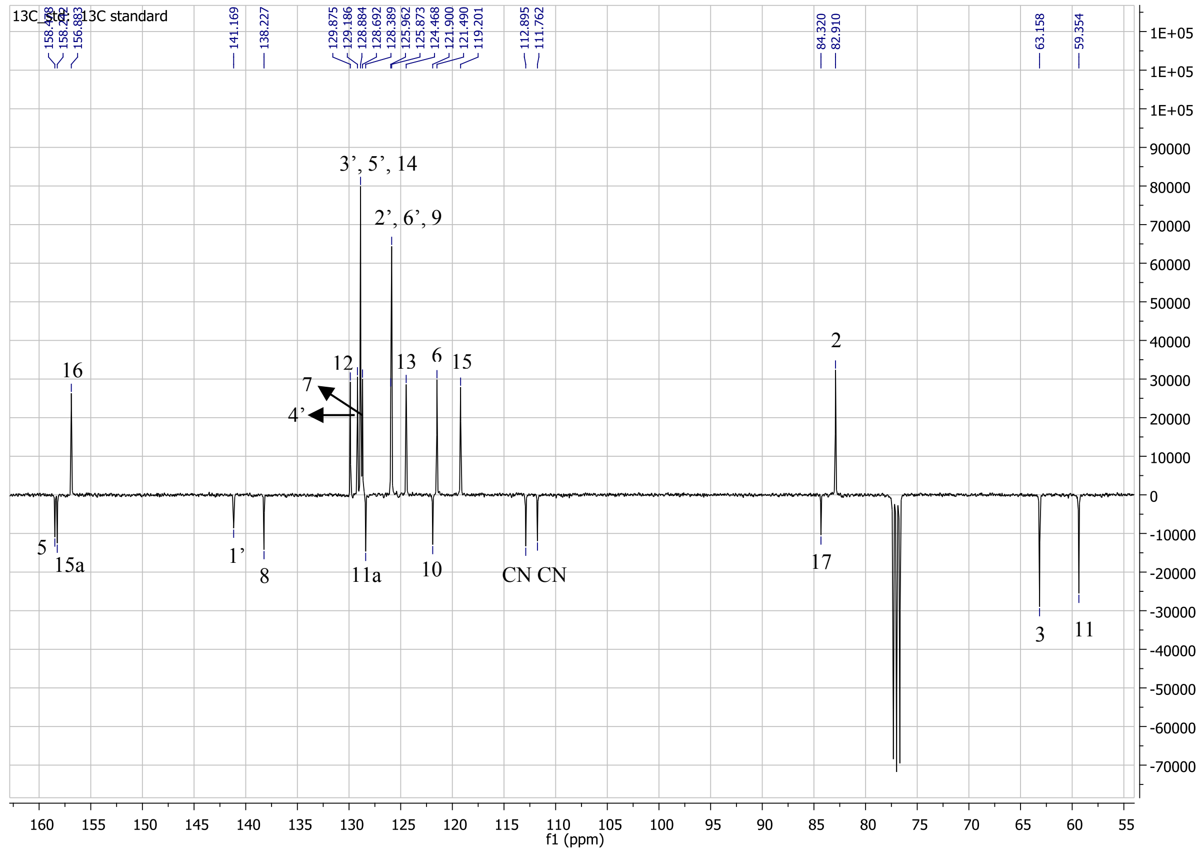

Figure S19. ^{13}C-NMR spectra of (*rac*)-**7c** measured in $CDCl_3$ 100 MHz.

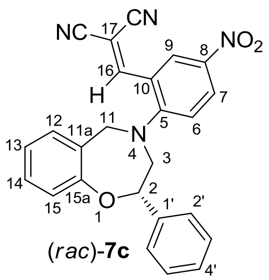

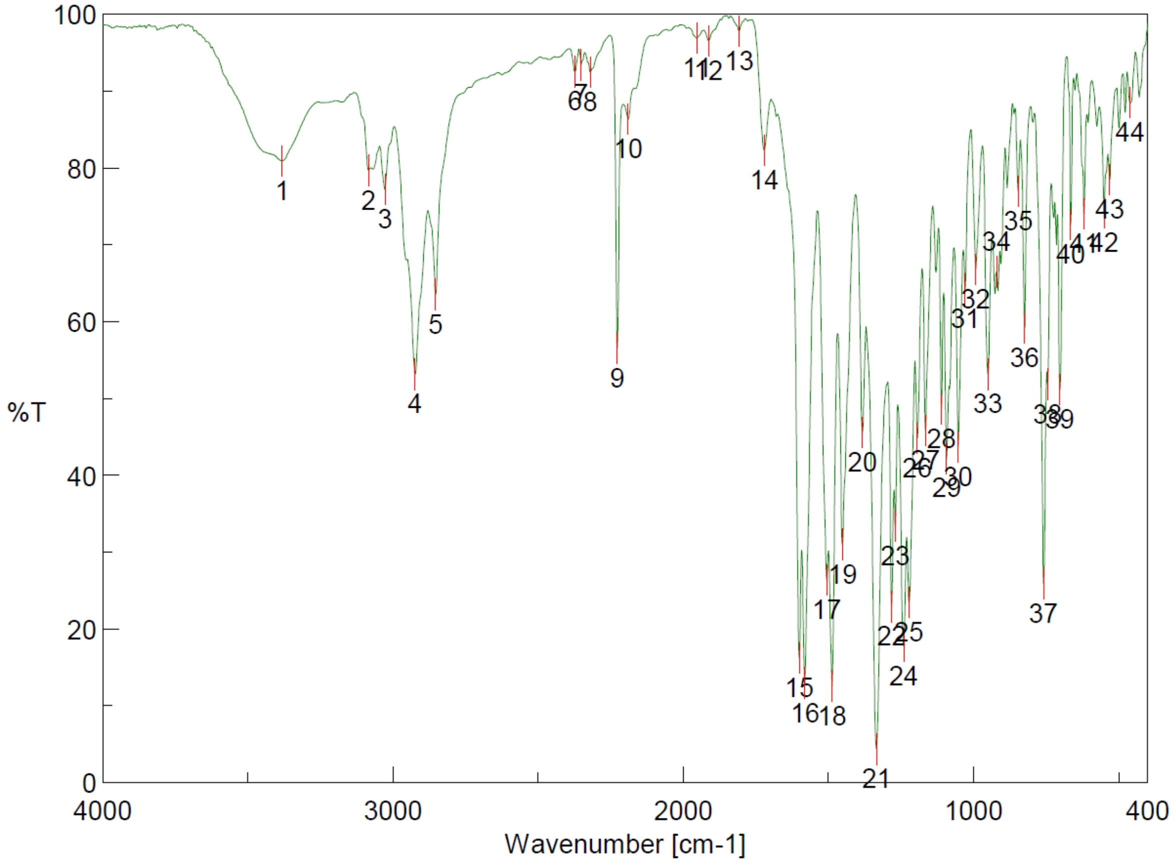

Figure S20. IR spectra of (*rac*)-**7c** recorded as KBr disc.
